# Tissue‐specific functions of MSCs are linked to homeostatic muscle maintenance and alter with aging

**DOI:** 10.1111/acel.14299

**Published:** 2024-09-25

**Authors:** Tamaki Kurosawa, Madoka Ikemoto‐Uezumi, Yuki Yoshimoto, Keitaro Minato, Noriyuki Kaji, Takashi Chaen, Eiji Hase, Takeo Minamikawa, Takeshi Yasui, Kazuhide Horiguchi, Satoshi Iino, Masatoshi Hori, Akiyoshi Uezumi

**Affiliations:** ^1^ Laboratory of Veterinary Pharmacology, Department of Veterinary Medical Sciences, Graduate School of Agriculture and Life Sciences Tokyo University Bunkyo‐ku Tokyo Japan; ^2^ Division of Cell Heterogeneity, Medical Research Center for High Depth Omics, Medical Institute of Bioregulation Kyushu University Fukuoka Japan; ^3^ Department of Molecular Craniofacial Embryology and Oral Histology, Graduate School of Medical and Dental Sciences Tokyo Medical and Dental University Bunkyo‐ku Tokyo Japan; ^4^ Division of Orthopedic Surgery, Department of Regenerative and Transplant Medicine, Graduate School of Medical and Dental Sciences Niigata University Niigata Japan; ^5^ Laboratory of Veterinary Pharmacology, School of Veterinary Medicine Azabu University Sagamihara Kanagawa Japan; ^6^ Division of Interdisciplinary Researches for Medicine and Photonics Institute of Post‐LED Photonics, Tokushima University Tokushima Japan; ^7^ Division of Next‐Generation Photonics Institute of Post‐LED Photonics, Tokushima University Tokushima Japan; ^8^ School of Health Sciences at Odawara International University of Health and Welfare Odawara Kanagawa Japan; ^9^ Department of Anatomy, Division of Medicine, Faculty of Medical Sciences University of Fukui Fukui Japan

**Keywords:** heterogeneity, keratocan, mesenchymal stromal cells, platelet‐derived growth factor receptor‐alpha, sarcopenia

## Abstract

Mesenchymal stromal cells (MSCs), also known as fibro‐adipogenic progenitors, play a critical role in muscle maintenance and sarcopenia development. Although analogous MSCs are present in various tissues, recent single‐cell RNA‐seq studies have revealed the inter‐tissue heterogeneity of MSCs. However, the functional significance of MSC heterogeneity and its role in aging remain unclear. Here, we investigated the properties of MSCs and their age‐related changes in seven mouse tissues through histological, cell culture, and genetic examinations. The tissue of origin had a greater impact on the MSC transcriptome than aging. By first analyzing age‐related changes, we found that *Kera* is exclusively expressed in muscle MSCs and significantly down‐regulated by aging. *Kera* knockout mice recapitulated some sarcopenic phenotypes including reduced muscle mass and specific force, revealing the functional importance of *Kera* in the maintenance of muscle youth. These results suggest that MSCs have tissue‐specific supportive functions and that deterioration in these functions may trigger tissue aging.

## Graphical abstract

In this study we revealed age‐related functional alterations specific to muscle MSCs. PDGFRα^+^ MSCs in young muscle specifically produce keratocan and contribute to the maintenance of muscle integrity by forming a healthy ECM. Aging alters the properties specific to muscle MSCs, thereby reducing keratocan production to disrupt the ECM structure. Age‐related alterations in muscle MSC‐specific functions can lead to sarcopenia.

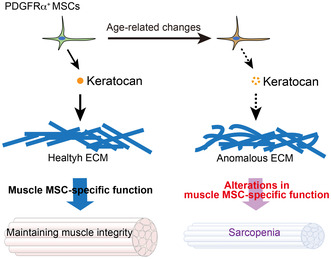

**Abbreviations**
bFGF: Basic fibroblast growth factor
DEGs: Differentially expressed genes
DMEM: Dulbecco's modified Eagle's medium
ECM: Extracellular matrix
FWHM: Full width at half maximum
GO: Gene ontology
MSCs: Mesenchymal stromal cells
PCA: principal component analysis
PDGFRα: Platelet‐derived growth factor receptor‐alpha
scRNA‐seq: Single‐cell RNA sequencing
SHG: Second harmonic generation
SLRP: Small leucine‐rich proteoglycan

## 1 INTRODUCTION

Skeletal muscles are the largest organ in the human body, accounting for approximately 30%–40% of the adult body weight. They primarily regulate bodily movement through contraction. In addition, skeletal muscles play a central role in systemic energy metabolism and produce bioactive substances to perform an endocrine‐like function, making it indispensable for the maintenance, and improvement of health. However, skeletal muscle function declines during aging, leading to sarcopenia.

Sarcopenia is defined as the loss of muscle mass and strength associated with aging. It can have adverse outcomes, including physical dysfunction, reduced quality of life, and even death (Cruz‐Jentoft et al., 2010). Sarcopenia is a major public health problem in modern society, affecting approximately 10%–16% of the elderly population worldwide (Yuan & Larsson, 2023). However, its etiology remains unclear. As the parenchymal cells of skeletal muscles are myofibers, aging changes in myofibers have been the primary focus in sarcopenia research. Although age‐related changes in myofibers undoubtedly shape the final phenotype of sarcopenia, there is limited scientific evidence that the onset of sarcopenia originates from age‐related changes in myofibers. In vivo and in vitro analyses of the contractile properties of muscles have shown that muscle strength declines with age, but the contractile capacity of isolated myofibers showed no differences between elderly and younger participants (Venturelli et al., 2015). Therefore, the quality of myofibers appears to be preserved even with aging. This suggests that factors other than myofibers, such as age‐related changes in the nervous system or extracellular matrix (ECM), may contribute to the development of sarcopenia (Grounds & Pinniger, 2015; Schüler et al., 2021; Venturelli et al., 2018).

To evaluate an element other than myofibers, the present study investigated Platelet‐derived growth factor receptor‐alpha (PDGFRα) positive cells in the muscle interstitium and their involvement in sarcopenia. In this paper, PDGFRα^+^ cells are referred to as “mesenchymal stromal cells” (MSCs), although they are sometimes called as fibroblasts, fibro‐adipogenic progenitors (FAPs), connective tissue fibroblasts, or mesenchymal stem cells. Our previous study successfully identified MSCs using PDGFRα as a marker, demonstrating that undesirable adipogenic differentiation of these cells leads to fatty degeneration of skeletal muscles (Uezumi et al., 2010). Fatty degeneration is a common age‐related change that is accompanied by muscle weakness (Delmonico et al., 2009). Genetically engineered mice lacking MSCs have shown a phenotype remarkably similar to sarcopenia, including loss of muscle mass and strength (Uezumi et al., 2021; Wosczyna et al., 2019). In addition, MSCs are the main source of the ECM affected during aging (Schüler et al., 2021), which could elucidate the etiology of sarcopenia. This suggests that the loss of normal MSC function triggers sarcopenia. PDGFRα also marks MSC/fibroblast populations in various other organs (Tabula Muris Consortium, 2018). Recent single‐cell RNA sequencing (scRNA‐seq) analyses have shown that MSCs in various tissues exhibit different gene expression profiles, revealing inter‐tissue heterogeneity in these cells (Muhl et al., 2020). However, the functional significance of MSC heterogeneity and its role in tissue aging remain unclear.

In this study, we performed comparative analysis of MSCs in seven mouse tissues (skeletal muscle, heart, white adipose tissue, lung, liver, intestinal mucosal layer, and intestinal muscle layer) between tissues and between young and aged mice. Differentiation assays and gene expression profiling revealed cross‐tissue heterogeneity and age‐related changes in MSCs. Further, integrated analysis of comparisons between tissues and between young and aged mice revealed a gene set that is specific to muscle MSCs and declines with age. We focused on one of these genes, *Kera*, and found that *Kera* is functionally important for maintaining muscle mass and specific force. Together, these findings provide the concept that MSCs have a supportive function specific to the tissues where they reside, and that dysregulation of these specific functions may trigger tissue aging.

## 2 RESULTS

### 2.1 In vivo visualization of MSCs in various tissue types

**FIGURE 1:**
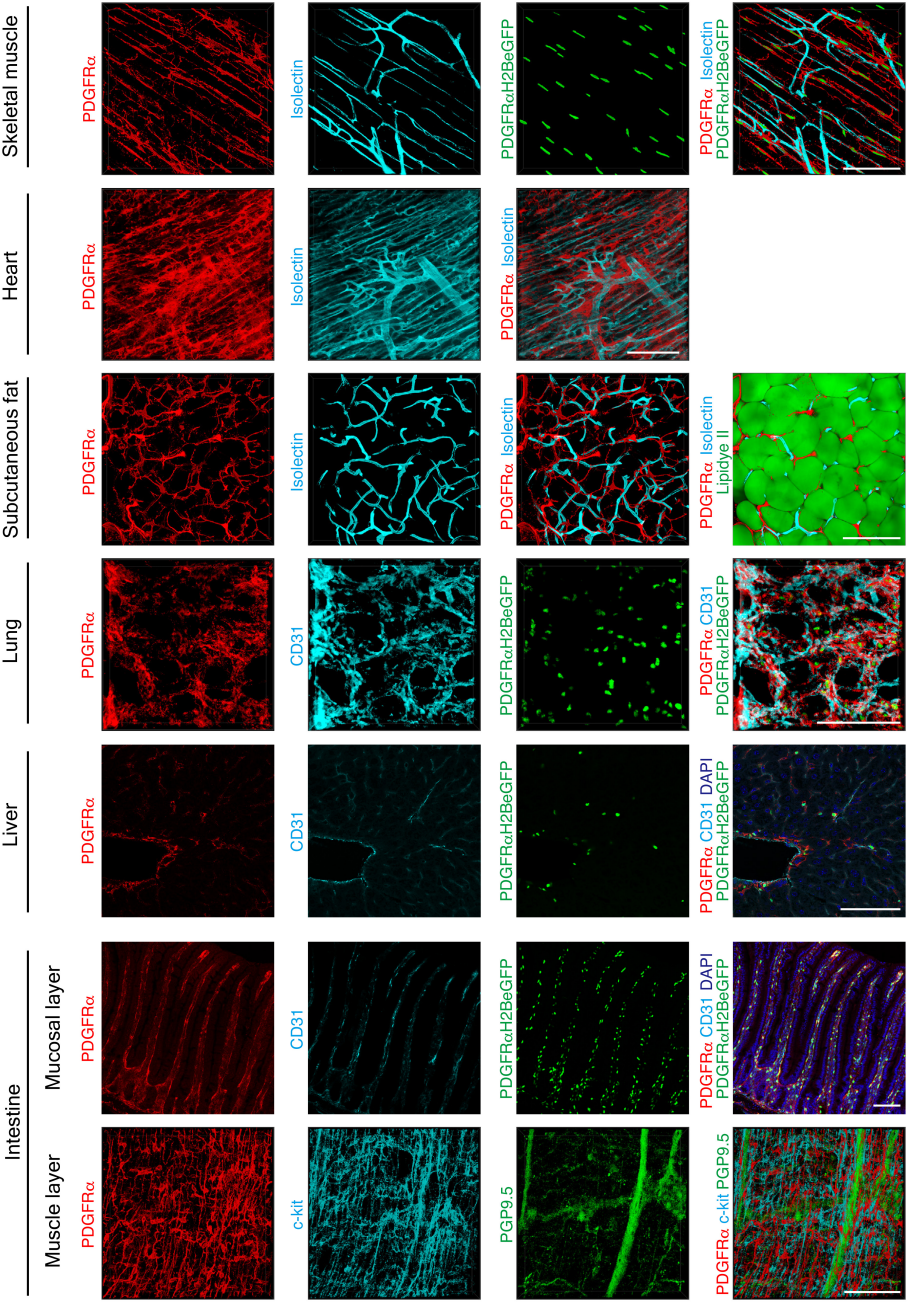
In vivo visualization of MSCs in various tissues of young mice. Fluorescence immunostaining images for PDGFRα^+^ cells in seven tissues of 3‐month‐old WT or PDGFRαH2BeGFP mice. Skeletal muscles (EDL: extensor digitorum longus), the heart, and subcutaneous fat were stained for PDGFRα (red) and Isolectin IB_4_ (light blue). Subcutaneous fat was stained with Lipidye II (green). The lungs, liver, and intestinal mucosal layer were stained for PDGFRα (red) and CD31 (light blue). The liver and intestinal mucosal layer were stained with DAPI (bule). The intestinal muscle layer was stained for PDGFRα (red), c‐kit (light blue), and PGP9.5 (green). Samples from PDGFRαH2BeGFP mice have nuclei of PDGFRα^+^ cells labeled with GFP (green). 3D images were obtained for whole‐mount staining of the skeletal muscles, heart, subcutaneous fat, lungs, and intestinal muscle layers. 2D images of frozen sections of the liver and intestinal mucosal layers were taken. Scale bar = 100 μm.

**FIGURE 2:**
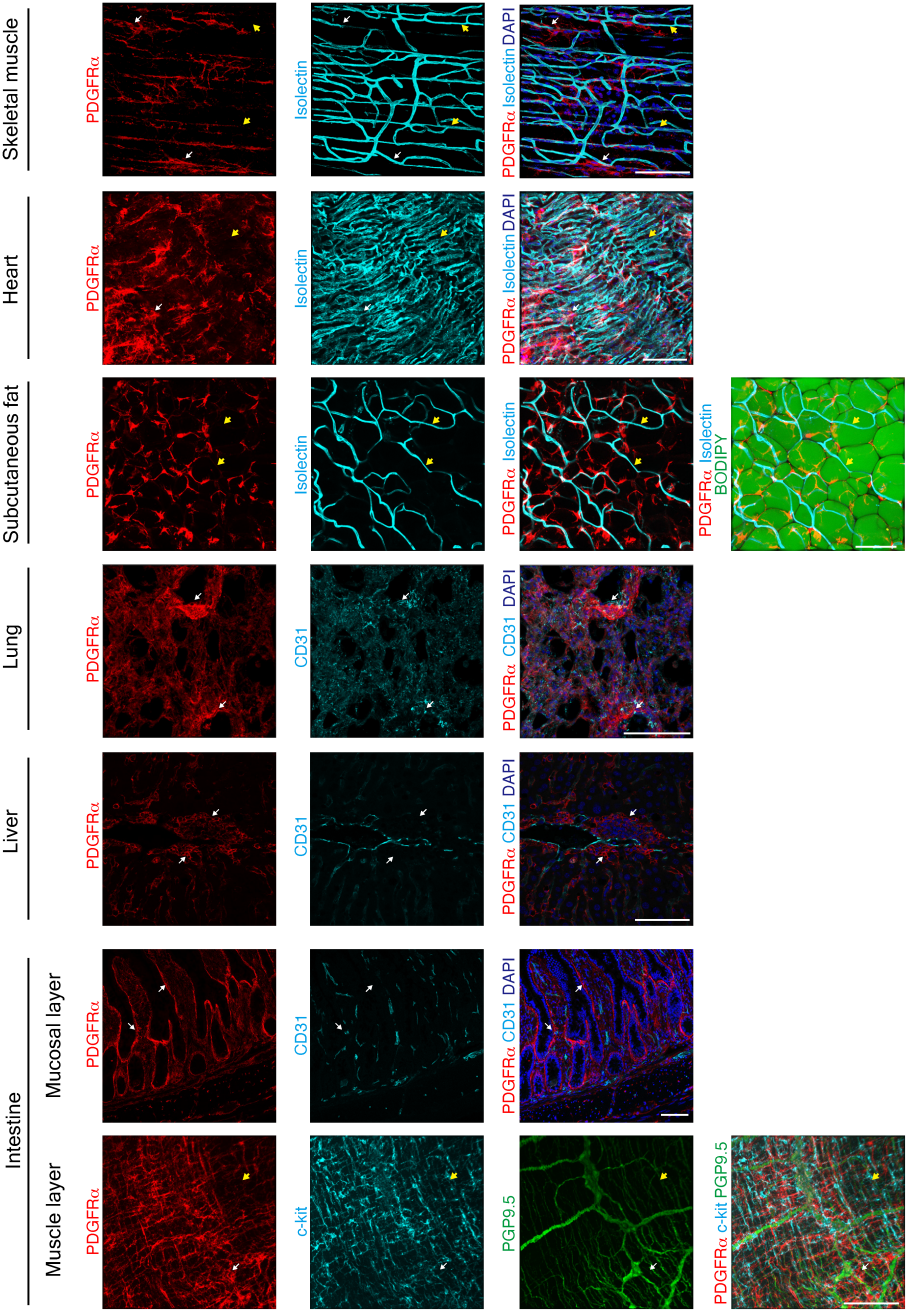
In vivo visualization of MSCs in various tissues of aged mice. Fluorescence immunostaining images for PDGFRα^+^ cells in seven tissue types of 28‐month‐old mice. The skeletal muscles, heart, and subcutaneous fat were stained for PDGFRα (red) and Isolectin IB_4_ (light blue). The subcutaneous fat was stained with BODIPY (green). The lungs, liver, and intestinal mucosal layer were stained for PDGFRα (red) and CD31 (light blue). The skeletal muscle, heart, lung, liver, and intestinal mucosal layers were stained with DAPI (blue). The intestinal muscle layer was stained for PDGFRα (red), c‐kit (light blue), and PGP9.5 (green). 3D images were obtained for whole‐mount staining of the skeletal muscle (EDL: Extensor digitorum longus), heart, subcutaneous fat, lungs, and intestinal muscle layers. 2D images of frozen sections of the liver and intestinal mucosal layers were taken. Yellow arrows point to areas with low densities of PDGFRα^+^ MSCs. White arrows point to areas with high densities of PDGFRα^+^ MSCs. Scale bar = 100 μm. [Correction added on 30 September 2024 after first online publication: Figure 1 had been inadvertently duplicated as Figure 2, and thus, Figure 2 has been corrected]

We analyzed age‐related changes in the MSCs of seven tissue types: the skeletal muscle, heart, subcutaneous fat, lung, liver, intestinal mucosal layer, and intestinal muscle layer. We first investigated the morphology and anatomical localization of MSCs in each tissue using PDGFRα as an MSC‐specific marker (Buechler et al., 2021; Muhl et al., 2020) in 3‐month‐old young mice. MSCs had a reticular morphology with projections in all tissues (Figure 1). MSCs appeared to be abundantly localized in the perivascular area of all tissues, at least in the area observed in this experiment, which is their typical anatomical localization (Benabid & Peduto, 2020). Consistent with previous findings (Kurahashi et al., 2020), MSCs, c‐kit‐positive intestinal cells of Cajal, and PGP9.5‐positive neurons in the intestinal muscle layer were present in close proximity (Figure 1). To investigate age‐related changes in MSC morphology and localization, we examined MSCs in seven tissue types from 28‐month‐old aging mice. MSCs appeared to be distributed throughout the tissue without significant distribution bias in young tissues, whereas very few (yellow arrows) or dense (white arrows) area of MSCs were observed in aged tissues in multiple location in multiple organs (Figure 2). A fluorescence‐activated cell sorting system (FACS) showed the proportion of MSCs present in each tissue, and it decreased with age in the liver and lung (Figure S1). As aging causes noticeable changes in the ECM, and MSCs are the main source of ECM production (Schüler et al., 2021; Statzer et al., 2023), the disorganized MSC distribution observed may indicate age‐related ECM dysregulation.

### 2.2 MSCs show various differentiation potentials depending on their tissue of origin

**FIGURE 3:**
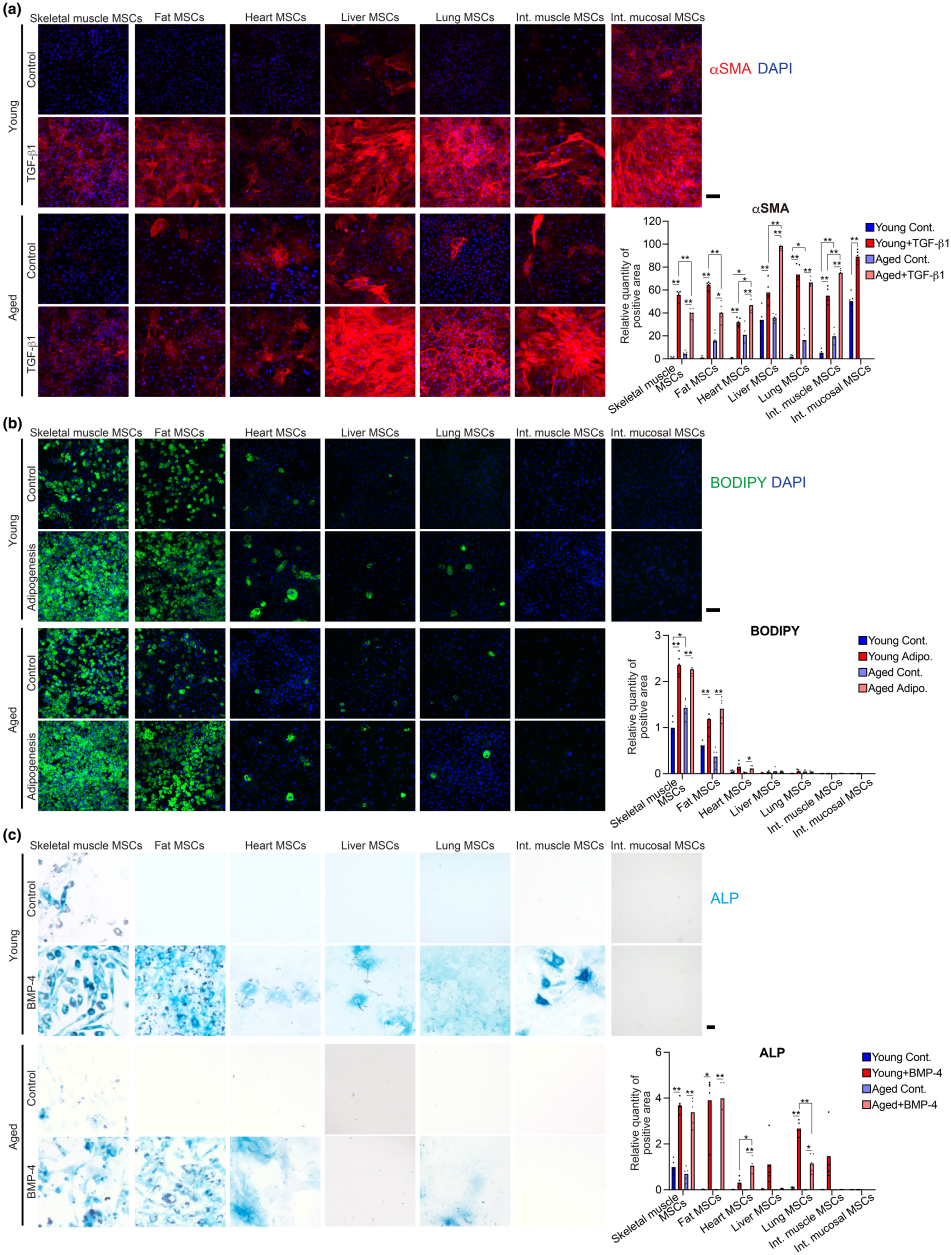
MSCs show varying differentiation potential depending on their tissue of origin. MSCs were sorted from seven tissues of young (3‐month‐old) and aged (28‐month‐old) mice to differentiate into fibrogenic cells, adipocytes, and osteoblasts in vitro. (a) Immunostaining images of MSCs for αSMA (red) and stained with DAPI (blue). MSCs were cultured with or without TGF‐β1. (b) BODIPY (green) and DAPI (blue) staining images of MSCs. (c) Alkaline phosphatase (ALP) (blue) staining images of MSCs. Data represent individual data points and the means; *n* = 5 areas were quantified for each group. Differences between the two groups were analyzed using a two‐sided unpaired *t* test. Differences between more than two groups were analyzed using one‐way analysis of variance (ANOVA) or Welch ANOVA tests when variances between the groups were not equal. **p < 0.01, *p < 0.05. Scale bar = 100 μm.

We investigated whether the differentiation potential of MSCs in different organs differs and how this is affected by aging. We analyzed the ability of MSCs to differentiate into fibrogenic cells, adipocytes, and osteoblasts in vitro. As MSCs from the intestinal mucosal layer of aged mice could not be cultured under the present conditions for unknown reasons, it was not possible to assess the differentiation potential of this population. MSCs from other tissues showed fibroblast‐like morphology in vitro (Figure S2). All MSCs differentiated into αSMA‐positive fibrogenic cells when treated with TGF‐β1 (Figure 3a), supporting reports indicating that they are the origin of fibrosis in various organs (Li et al., 2018; Ramachandran et al., 2019; Uezumi et al., 2011). For adipogenesis, MSCs were cultured with induction medium (DMEM supplemented with 10 μg/mL insulin, 0.25 μM dexamethasone, 0.5 mM IBMX, and 10% FBS) for 3 days and with maintenance medium (DMEM supplemented with 10 μg/mL insulin and 10% FBS) for 4 days until fat droplets increased. MSCs of the skeletal muscle and subcutaneous fat spontaneously differentiated into adipocytes, even in the control medium (DMEM supplemented with 10% FBS). Additionally, they differentiated into adipocytes with large lipid droplets when induced in adipogenic medium (Figure 3b). In contrast, MSCs from the heart, liver, and lungs produced only a few adipocytes, whereas those from the intestines produced no adipocytes (Figure 3b). MSCs of the skeletal muscle, subcutaneous fat, and lungs produced abundant alkaline phosphatase‐positive osteogenic cells. Contrastingly, MSCs of the heart, liver, and intestinal muscle layer produced only a few osteogenic cells after BMP‐4 induction. The MSCs in the intestinal mucosal layer did not produce osteogenic cells (Figure 3c). These results suggest that MSC cellular properties are dependent on their tissue of origin. MSCs of the heart, liver, and intestinal muscle layer from aged mice showed higher differentiation potential into αSMA‐positive cells than those from young mice under TGF‐β1 induction (Figure 3a). In addition, MSCs of the heart, lung, and intestinal muscle layer from aged mice spontaneously differentiated into αSMA‐positive cells even in the control medium (Figure 3a). Furthermore, MSCs from the skeletal muscle of aged mice showed more robust spontaneous differentiation into adipocytes when cultured in the control medium than those from young mice (Figure 3b). These results indicate an age‐related alteration in the differentiation properties of MSCs, which may be related to the increased susceptibility of tissues to fatty degeneration and fibrosis during aging (Marcus et al., 2010; Selman & Pardo, 2021).

### 2.3 Transcriptome analysis reveals the tissue‐specific gene signature of MSCs

**FIGURE 4:**
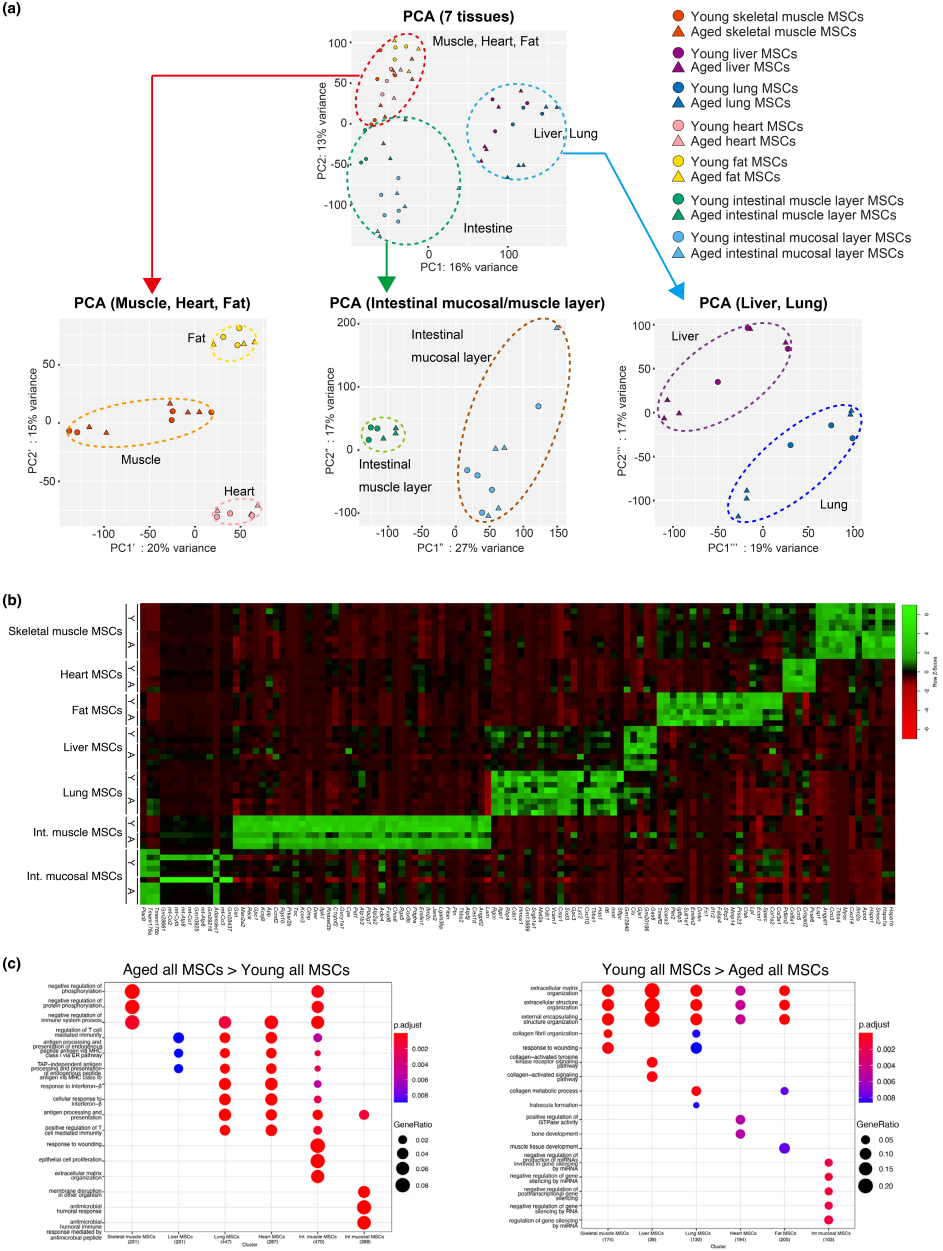
Transcriptome analysis of MSCs reveals the tissue‐specific gene signature. MSCs were sorted from seven tissues of young (3‐month‐old) and aged (28‐month‐old) mice and analyzed by bulk RNA‐seq. (a) PCA analysis for RNA‐seq of MSCs from seven tissue types of young and aged mice. *n* = 5 mice for young skeletal muscle MSCs; *n* = 5 for aged skeletal muscle MSCs; *n* = 3 for young heart MSCs; *n* = 3 for aged heart MSCs; *n* = 3 for fat MSCs; *n* = 3 for aged fat MSCs; *n* = 3 for young liver MSCs; *n* = 5 for aged liver MSCs; *n* = 3 for young lung MSCs; *n* = 5 for aged lung MSCs; *n* = 3 for young intestinal muscle layer MSCs; *n* = 3 for aged intestinal muscle layer MSCs; *n* = 5 for young intestinal mucosal layer MSCs; and *n* = 5 for aged intestinal mucosal layer MSCs. The dashed circles represent parental clusters (upper column) and subclusters separated by tissue of origin (lower column). (b) This heat map compares gene expression levels relative to each tissue MSC of the young (Y) and aged (A) groups to visualize tissue specificity of MSCs. Genes with TPM values >150 and expression ratios >1 in log2FC to those in MSCs from other tissues' MSCs were selected as genes that refract MSC tissue specificity. The color scale value is based on Z‐score. (c) GO terms enriched in the differentially expressed genes (DEGs) in MSCs from seven tissue types of young and aged mice. The genes with a variation of BaseMean >100, log2FC >1, and adj. *p* < 0.05 were selected as DEGs.

To elucidate organ specificity and age‐related changes in MSCs, we performed RNA‐seq on MSCs from seven tissues of young and aged mice. MSCs were roughly divided into three clusters during principal component analysis (PCA): clusters consisting of the muscle, heart, and fat; the liver and lung; and the intestine (Figure 4a). These were clearly divided into subclusters depending on the tissue of origin when PCA was performed on each cluster (Figure 4a). Young and aged MSCs fell into the same subcluster, even when PCA was performed against each parental cluster (Figure 4a). When PCA was performed on individual tissues, separate clusters of young and aged appeared in some tissues (Figure S3). These results indicate that the influence of the tissue of origin on the properties of MSCs characterized at least by the whole transcriptome was stronger than that of aging. The tissue‐specific nature of MSCs is also shown by a heat map (Figure 4b and Table S1).

Age‐related changes common to MSCs from several tissues were observed. Gene ontology (GO) analysis revealed elevated gene expression related to inflammatory and immune responses in the MSCs of six aged tissues (Figure 4c left, and Figure S4). Immunostaining revealed abundant CD45‐positive immune cells closely localized to MSCs in aged tissues, suggesting an age‐related increase in cell–cell interactions between immune cells and MSCs (Figure S5). In contrast, decreased gene expression related to ECM and ECM‐degrading enzymes was common to the MSCs of five aged tissue types, suggesting reduced ECM turnover in aged tissues (Figure 4c right and Figure S4). Increased inflammation and reduced ECM turnover are consistent with age‐related changes in tissues (Gomes et al., 2021; Podolsky et al., 2020).

### 2.4 *Kera* is a gene unique to muscle MSCs, and its expression decreases with age

**FIGURE 5:**
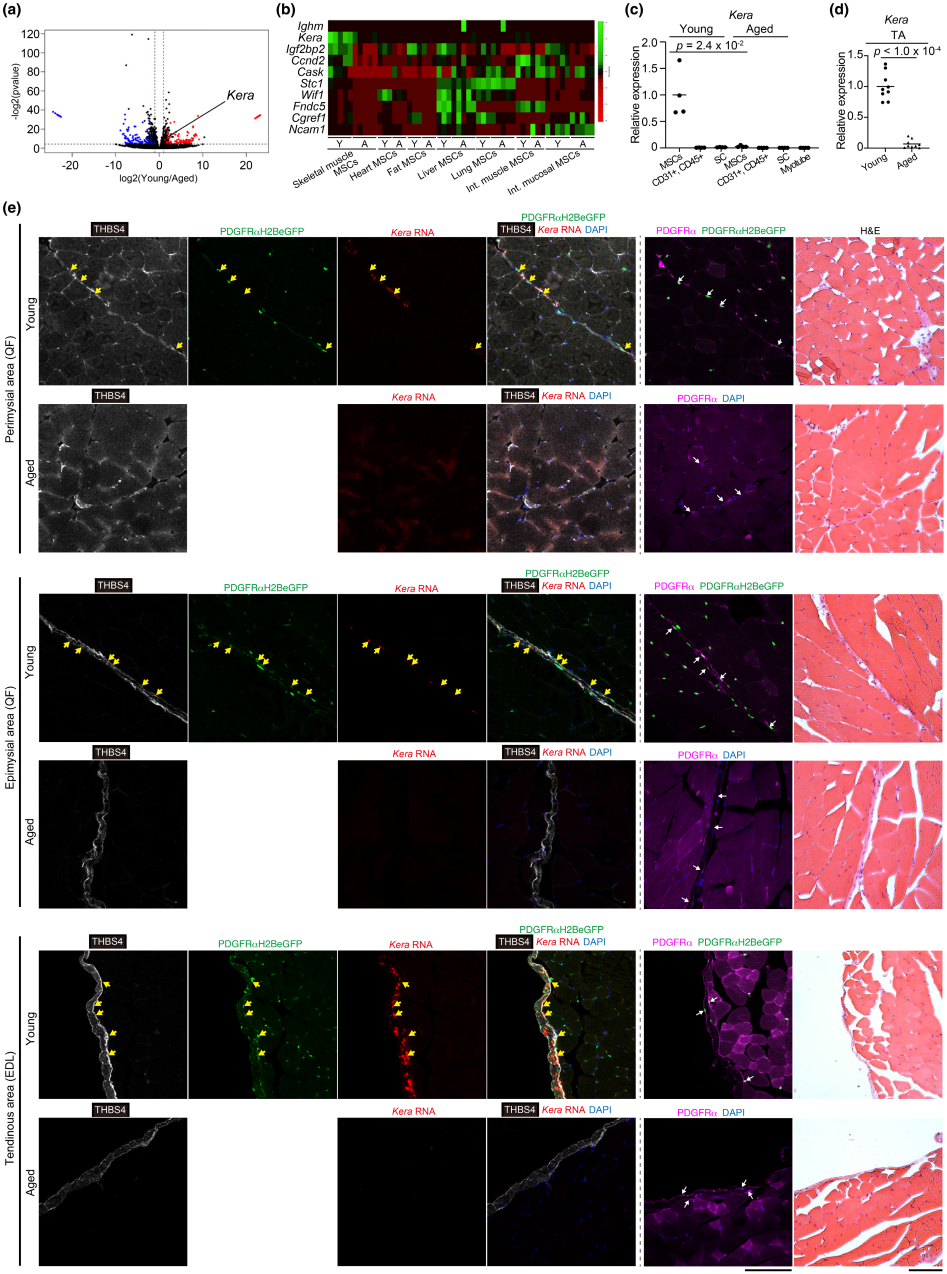
*Kera* is exclusively expressed in muscle MSCs and is significantly reduced by aging. (a) A volcano plot of DEGs in young and aged muscle MSCs. *n* = 5 mice for both young and aged groups. The genes with variations of BaseMean >100, log2FC >1, and adj. *p* < 0.05 were selected as DEGs. The red and blue dots represent the genes whose expression is significantly downregulated and upregulated, respectively, with aging. (b) This heat map shows the expression levels of the 10 genes with the highest TPM in young muscle MSCs among genes that significantly decreased with the aging of muscle MSCs in each tissue MSC of the young (Y) and aged (A) groups. The genes with variations of log2FC >2.5 and adj. *p* < 0.05 were selected as DEGs. The samples shown in Figure 4 were used for this analysis. (c) The quantified expression levels of *Kera* in sorted cells. MSCs: PDGFRα‐positive MSCs, CD31+ CD45+: CD31, or CD45‐positive cells, SC: Satellite cells. Myotubes were differentiated from sorted satellite cells. *n* = 4 mice. (d) The quantified expression levels of *Kera* in the tibialis anterior (TA) muscles of young or aged mice. *n* = 9 mice for both young and aged groups. The data represent individual data points and the means. Data were analyzed using a two‐sided unpaired *t* test (c, d). (e) In situ hybridization on muscle sections from 10 weeks of PDGFRαH2BeGFP mice (young) and 24‐month‐old WT mice (aged) with *Kera* probe (red) followed with immunostaining to THBS4 (white) and GFP (green) and stained with DAPI (blue). Images of serial sections stained with PDGFRα antibody (magenta) followed with H&E staining are shown in the same row to the right side of the dashed lines. Yellow arrows indicate *Kera*+/PDGFRαH2BeGFP+ nuclei. White arrows indicate PDGFRα+ cells. QF: Quadriceps femoris, EDL: Extensor digitorum longus. Scale bar = 100 μm.

PDGFRαCreER/R26‐DTA mice lacking MSCs developed muscle atrophy and weakness in previous studies (Uezumi et al., 2021; Wosczyna et al., 2019), demonstrating the essential role of MSCs in the maintenance of muscle tissue. In this study, we attempted to identify age‐related changes that contribute to sarcopenia in muscle MSCs. Gene expression in the muscle MSCs of young and aged mice was compared using DEseq2 (Figure 5a and Tables S2 and S3). We searched for genes whose expression was significantly downregulated during aging (>2.5 log2FC) and listed the 10 genes with the highest expression levels in young muscle MSCs (Figure 5b). *Kera*, a gene that encodes keratocan (Liu et al., 2003), was expressed only in muscle MSCs but not in MSCs from other tissues (Figure 5b and Table S4). Quantitative PCR revealed that *Kera* expression was highly exclusive to MSCs and undetectable in other cell types within the muscle tissue (Figure 5c and Figure S6). *Kera* expression significantly decreased with age in both sorted cells (Figure 5c) and muscle tissue (Figure 5d). Thus, *Kera* is a gene exclusively expressed in muscle MSCs, and its expression considerably diminishes with age.

We examined the localization of *Kera* expression in skeletal muscle through in situ hybridization and found that *Kera* was expressed in the perimysium as previously reported (Figure 5e) (Muhl et al., 2020). In addition, *Kera* was found to be expressed in the epimysial and tendinous areas (Figure 5e and Figure S7a). We noticed that *Kera* is expressed not only in tendon per se, but also in many PDGFRα^+^ MSCs localized at the myotendinous junction, MTJ (Figure 5e and Figure S7a). The percentage of *Kera*‐expressing MSCs to total MSCs was 8.6 ± 1.3%, indicating that *Kera* is expressed in a specific subset of MSCs (Figure S7b). The restricted expression of this gene demonstrates the intra‐tissue heterogeneity of MSCs (Muhl et al., 2020; Uapinyoying et al., 2023). *Kera* expression in these areas was almost completely absent in the muscles of aged mice (Figure 5e).

### 2.5 *Kera* KO mice lose muscle mass and strength even at a young age

**FIGURE 6:**
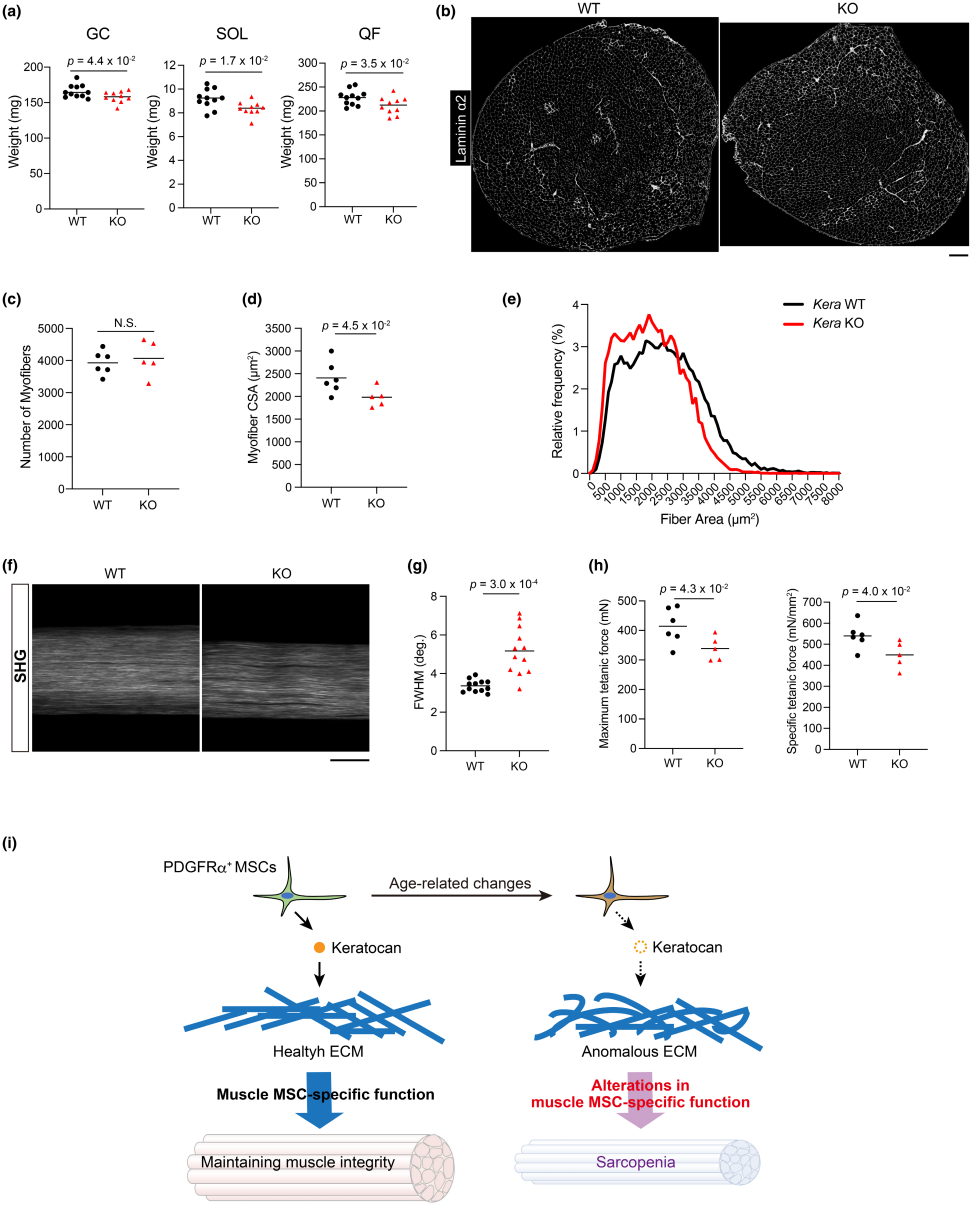
*Kera* KO mice show the loss of muscle mass and strength even at a young age. (a). The muscle weight of the male *Kera* KO or wildtype (WT) littermates was measured at 10 weeks of age. GC: Gastrocnemius, QF: Quadriceps femoris, SOL: Soleus. *n* = 10 (*Kera* KO) and *n* = 11 (WT). (b). QF cross sections stained for laminin α2. (c–e). The number of myofibers (c), the myofiber cross‐sectional area (CSA) (d), and myofiber CSA distribution (e) in QF muscles; *n* = 5 (*Kera* KO) and *n* = 6 (WT). (f). Second harmonic generation (SHG) images for extensor digitorum longus tendons of the *Kera* KO or WT littermates. (g). Analysis of the full width at half maximum (FWHM) of the SHG angular spectrum; *n* = 12 mice per group. (h). Measurement of the maximum and specific tetanic force of the EDLs; *n* = 5 (*Kera* KO) and *n* = 6 (WT). The data represent individual data points and the means. Data were analyzed using a two‐sided unpaired *t* test (a, c, d, g, h). Scale bar = 300 μm for B and 50 μm for F. (i). A model in which muscle MSCs maintain youthfulness of skeletal muscles by specifically producing keratocan. PDGFRα^+^ MSCs in young muscle specifically produce keratocan and contribute to the maintenance of muscle integrity by forming a healthy ECM. Aging alters the properties specific to muscle MSCs, thereby reducing keratocan production to disrupt the ECM structure. Age‐related alterations in muscle MSC‐specific functions can lead to sarcopenia.

We analyzed the skeletal muscle of *Kera* knockout (*Kera* KO) mice (Liu et al., 2003) to investigate the physiological function of keratocan in muscles. *Kera* expression was completely lost in the perimysium, epimysium, and tendons of *Kera* KO mice (Figure S8). There is no difference in muscle weight between 5‐week‐old WT and *Kera* KO mice in both males and females (Figure S9a). However, the weights of the gastrocnemius, soleus, and quadriceps femoris muscles were all reduced in 10‐week‐old male *Kera* KO mice, but not in female mice (Figure 6a and Figure S9b). These data indicate that *Kera* deficiency does not affect muscle development and formation in both male and female mice, but affects the muscle phenotype over time after normal muscle formation in male. Given that age‐related decline in skeletal muscle mass have been reported to be greater in men than women (Thomas, 2007), it is interesting to assume that the sex differences observed in *Kera* KO mice are related to sex differences in muscle aging. Thus, we focused on phenotypes of male mice for subsequent analyses. The reduced muscle mass in *Kera* KO mice was not due to changes in the number of myofibers. Rather, it was due to their atrophy, as evidenced by a decrease in the cross‐sectional area (Figure 6b–e). Loss of *Kera* expression did not alter the number of PDGFRα^+^ MSCs in skeletal muscle tissue (Figure S10). As keratocan belongs to small leucine‐rich proteoglycans (SLRPs) and SLRPs are known to contributes to collagen fibrillogenesis (Kalamajski & Oldberg, 2010), we examined the collagen structure of the tendon of *Kera* KO mice using second harmonic generation (SHG) microscope capable of analyzing the distribution and orientation of collagen fiber (Figure 6f) The full width at half maximum (FWHM) of the SHG angular spectrum obtained using 2D Fourier transform (2D‐FT) analysis (Figure 6f) was significantly increased in *Kera* KO mice (Figure 6g), indicating that *Kera* deficiency altered collagen fiber orientation. Tendons and interstitial ECM are continuous integral structures essential for force transmission in skeletal muscle (Huijing, 1999). Analysis of the contractile properties of skeletal muscles in *Kera* KO mice revealed a significantly reduced maximum and specific tetanic forces (Figure 6h). This suggests that the reduced muscle force in *Kera* KO mice is not simply the result of a loss of muscle mass but is due to a deterioration in the intrinsic quality of the muscle tissue. These results indicate that keratocan plays a critical role in the maintenance of muscle mass and strength.

## 3 DISCUSSION

This study revealed the cross‐tissue heterogeneity of MSCs and identified keratocan as a functional molecule specific to muscle MSCs. The present study further demonstrated that keratocan expression is downregulated with age. *Kera* KO mice showed no difference in muscle mass compared to WT in both males and females at 5 weeks of age, indicating that *Kera* deficiency does not affect normal muscle development and formation. At 10 weeks of age, male *Kera* KO mice exhibit reduced muscle mass and specific force, as well as disturbed collagen orientation even at an early age. No muscle loss was observed in 10‐week‐old female *Kera* KO mice, which may reflect a sex difference in muscle aging, where age‐related loss of muscle mass is greater in men than women (Thomas, 2007). Female KO mice may show a muscle phenotype with longer‐term observation, but this was not pursued here and is a limitation of this study. Because *Kera* expression is almost completely lost with age, it is highly possible that aging induces abnormalities similar to those observed in the *Kera* KO mice. Thus, we propose a model in which age‐related alterations in muscle MSC‐specific functions lead to sarcopenia, which is a muscle‐specific aging phenomenon (Figure 6i).

In this study, we analyzed MSCs in various organs using PDGFRα as a marker. PDGFRα^+^ cells contain a cell population called mesenchymal stem cells (Miwa & Era, 2018). However, the stemness of mesenchymal stem cells has not been convincingly proven, and it is not appropriate to call these cells “stem cells” (Caplan, 2017). Therefore, the term “stem cells” was avoided, and “mesenchymal stromal cells” was used in this study. MSCs by nature exhibit a degree of plasticity. Although the effects of aging on MSC plasticity are not completely understood, we found that MSCs from heart, lung, and intestinal muscle layer of aged mice and MSCs from the skeletal muscle of aged mice are prone to spontaneous differentiation into fibrogenic cells and adipocytes, respectively. Therefore, MSC plasticity is likely to be affected by aging, which may be involved in age‐related tissue deterioration such as fatty degeneration and fibrosis.

Muscle strength is considered a more important indicator of sarcopenia because its reduction is more strongly associated with adverse outcomes than that of muscle mass (Cruz‐Jentoft et al., 2019). Specific force is the maximal force normalized on the basis of physiological cross‐sectional area. A decline in the specific force of muscle tissue is the primary contributor to age‐related muscle weakness (Morse et al., 2005). However, the mechanism underlying specific force decline remains unclear. The ECM of skeletal muscles is essential to defining specific force because it plays a muscle tissue‐specific role in transmitting the contractile force generated by muscle fibers (Azizi et al., 2017; Sharafi & Blemker, 2011). However, it is markedly altered by aging. Comprehensive gene expression analysis of the aging process in muscle tissue has revealed changes in the characteristics of muscle tissue aging, including decreased ECM remodeling (Ham et al., 2020). A proteomic study has revealed significant age‐related ECM changes in muscle tissue and identified MSCs as major contributors to age‐dependent changes in the skeletal muscle ECM (Schüler et al., 2021). These comprehensive analyses of muscle tissue also showed decreased *Kera* expression with age, further supporting the present results.

*Kera* is an ECM component belonging to SLRP family, and SLRPs play regulatory roles in collagen fibrillogenesis and ECM assembly (Chen & Birk, 2013). The deletion of *Kera* causes structural abnormalities in the cornea (Liu et al., 2003), but its function in the musculoskeletal system is unknown. This study showed that *Kera* is highly expressed in the perimysium, epimysium, MTJ, and tendons, and that *Kera* deficiency results in decreased specific force of muscle tissue. These results suggest that *Kera* contributes to muscle force production by regulating ECM function in areas important for force transmission, such as the perimysium, epimysium, MTJ, and tendons. Additionally, these findings identify reduced *Kera* expression as a potential factor in age‐related muscle weakness. It should be mentioned that we only analyzed a full‐body knockout of *Kera*. Although our qPCR and in situ hybridization analyses showed that *Kera* is specifically expressed in MSCs, low‐level expression of *Kera* in other cell types including cells outside skeletal muscle possibly contribute to the phenotypes of *Kera* KO mice. The ideal way to demonstrate the functional importance of *Kera* in MSCs would be to use cell type‐selective conditional KO mice, but this has not been done here, which is a limitation of this study.

Cellular senescence with high p16 expression in skeletal muscles occurs exclusively in PDGFRα^+^ cells (Zhang et al., 2022). The importance of the stroma cells in systemic aging is also becoming clear. Mechanosignaling (YAP/TAZ activity) selectively declines during aging in stromal cells, which mainly comprise MSCs residing in various tissues. Furthermore, reduced mechanosignaling in stromal cells accelerates cellular senescence and tissue aging (Sladitschek‐Martens et al., 2022). We previously showed that MSCs in the muscle stroma are essential for the maintenance of steady‐state muscles (Uezumi et al., 2021). The present study revealed that *Kera* is expressed by MSCs and that its age‐related decreased expression levels reduce muscle function. Therefore, we propose a generalized model of aging that can be applied to various tissues in which age‐related changes in MSCs initiate and accelerate tissue aging.

In this study, we reveled the functional significance of the inter‐tissue heterogeneity of MSCs. Meanwhile, it is known that there is heterogeneity in MSC gene expression even within a single tissue. Scott et al. and Oprescu et al. showed that MSCs from intact mouse skeletal muscles are composed of two distinct subpopulations, one characterized by *Dpp4* expression and another by *Cxcl14* expression (Oprescu et al., 2020; Scott et al., 2019). Muhl et al. (2020) performed scRNA‐seq on mesenchymal cells including fibroblasts and mural cells derived from four different mouse tissues and identified 10 subpopulations within skeletal muscle. Their database showed that *Kera* was exclusively expressed in perimysial cells of skeletal muscle, which is consistent with our findings. The functional significance of the heterogeneity of gene expression profiles in MSCs revealed by previous scRNA‐seq studies remains unclear. However, our findings demonstrate that *Kera*‐expressing MSCs localized to the epimysium, perimysium, and MTJ have unique functions and contribute to the maintenance of muscle mass and strength. This suggests that each MSC subpopulation may have distinct functions in tissue maintenance.

In summary, our results confer functional significance to MSC heterogeneity and demonstrate its importance in tissue aging. However, further studies are needed to elucidate the functions of individual MSC subpopulations, which can provide additional insight into skeletal muscle maintenance and aging.

## 4 METHODS

### 4.1 Mice

C57BL/6 mice were purchased from Japan SLC (Shizuoka, Japan). PDGFRαH2BeGFP (stock# 007669) and *Kera* KO (stock# 025568) mice were purchased from Jackson Laboratory (Bar Harbor, ME, USA). The sexes of the mice used in the analysis are listed in Table S5. The mice were supplied with food and water ad libitum and kept under constant temperature and humidity in a 12‐h dark/light cycle. All animal experiments were performed in accordance with the ARRIVE guidelines. All the animal experiments were approved by the Experimental Animal Care and Use Committee of the University of Tokyo.

### 4.2 Histology

The mice were euthanized through cervical dislocation, and tissues were collected. The lungs, heart, and subcutaneous fat were fixed in 4% paraformaldehyde (PFA) for 30 min, washed three times with PBS, and cut into thin pieces of <1 mm for whole‐mount staining. The mucosal layers were discarded to stain the intestinal muscle layer. The muscle layers were then pinned onto a silicon dish, fixed with ice‐cold acetone for 30 min, and washed three times with PBS. To transparency and stain the whole tissue, extensor digitorum longus muscle (EDL), which are of a suitable size for 3D observation, were used as skeletal muscle samples. The muscle as extracted by cutting both ends of the tendons, clamped onto a silicone dish, fixed with 4% PFA using an intradermal needle (SEIRIN Corp., Shizuoka, Japan) for 30 min, and washed with PBS three times. All tissue samples were incubated with 1% triton and 4% BSA in PBS overnight at 4°C. Next, samples were incubated with the primary antibodies overnight at 4°C, followed by incubation with the secondary antibodies overnight at 4°C. Tissue samples underwent clearing with 60% 2,2’‐Thiodiethanol (Sigma‐Aldrich, St. Louis, MO, USA) in PBS solution for 30 min before microscopic observation. The freshly frozen liver and intestinal tissues were sectioned into 10‐μm thick sections using a cryostat (Leica Microsystems, Wetzlar, Germany) for the immunostaining of cross sections. The sections were fixed with 4% PFA for 5 min and blocked with Protein Block Serum‐Free (Agilent Technologies, Santa Clara, CA, USA) for 5 min. Sectioned samples were incubated with primary antibodies overnight at 4°C, followed by incubation with secondary antibodies for 1 h. Sections were mounted using SlowFade Diamond Antifade Reagent (Thermo Fisher Scientific, Waltham, MA, USA). Fluorescent images were captured using the DM6000FS fluorescence microscope (Leica Microsystems). Confocal images were captured using a confocal laser scanning microscopy system: TCS SP8 (Leica Microsystems) or A1Rsi (Nikon, Tokyo, Japan). To analyze the muscle fiber cross‐sectional area (CSA), 10‐μm thick frozen sections were cut 4 mm from the proximal end of the gastrocnemius muscle. Sections were fixed in ice‐cold acetone for 5 min and incubated with primary antibodies at 4°C overnight, followed by incubation with the secondary antibody. Images of the entire sections were captured using the BZ‐X710 fluorescence microscope (Keyence, Osaka, Japan). CSA and myofiber were quantified using the Hybrid Cell Count software (Keyence). The primary and secondary antibodies used for staining are listed in Table S6.

### 4.3 In situ hybridization

Skeletal muscles for in situ hybridization were fixed in 4% PFA for 30 min. Muscles were equilibrated in a PBS solution containing 20% sucrose, frozen in Optimal Cutting Temperature compound, and sectioned into 10‐μm thick sections. In situ hybridization was performed using the RNAscope 2.5 HD Reagent Kit (#322350; Advanced Cell Diagnostics, Inc.; ADC, Newark, CA, USA) and the RNAscope probe Mm‐*Kera* (#562351; ADC). The sections were stained with the primary antibodies at 4°C overnight after in situ hybridization, followed by incubation with the secondary antibodies and DAPI (DOJINDO, Kumamoto, Japan), listed in Table S6.

### 4.4 RNA extraction and real‐time PCR analysis

Freshly frozen tibialis anterior muscles and sorted cells were used to extract RNA. Muscles were smashed with 3 mm zirconia beads in QIAzol Lysis Reagent (Qiagen, Hilden, Germany) using a Shakeman homogenizer (Bio Medical Science, Tokyo, Japan). Total RNA was extracted using an RNeasy Mini Kit (Qiagen). The extracted RNA was reverse‐transcribed into cDNA using a QuantiTect Reverse Transcription Kit (Qiagen). Real‐time PCR analysis was performed using the Takara Thermal Cycler Dice Real‐Time System (Takara Bio, Shiga, Japan) with SYBR Premix Ex Taq II (Takara Bio). The PCR conditions were as follows: 95°C for 60 s, followed by 40 cycles of 95°C for 15 s, 60°C for 20 s, and 72°C for 30 s for tissue samples. The relative expression levels of the target genes were calculated using the ΔΔCt method. *Hsbp1* or *Cmas* were used as reference genes to analyze target gene expression levels. The primers used for PCR are listed in Table S7.

### 4.5 Cell isolation

#### 4.5.1 Skeletal muscle

Tissue digestion was performed as previously described (Uezumi et al., 2021). Minced whole hind limb muscles from two mice were digested with 0.2% type II collagenase (Worthington, Lakewood, NJ, USA) for 60 min at 37°C. Digested muscles were passed through an 18‐G needle five times and further digested for 30 min at 37°C.

#### 4.5.2 Heart

Tissue digestion was performed as previously described (Uezumi et al., 2021). The atrium was removed and each ventricle was finely minced. Minced hearts were digested with 0.2% type II collagenase (Worthington) for 5 min at 37°C. The digested hearts were passed through an 18‐G needle several times. Digestion and needle homogenization were repeated three times.

#### 4.5.3 Subcutaneous fat

The inguinal lymph nodes were removed, and each side of the subcutaneous fat was finely minced. Minced fats were digested with 0.2% type II collagenase (Worthington) for 30 min at 37°C. Digested fats were passed through an 18‐G needle several times and further digested for 10 min at 37°C.

#### 4.5.4 Liver

All surgical incisions were made under deep anesthesia. The right atrium was incised with dissecting scissors, and a 23‐G needle was inserted into the left ventricle. Next, 20 mL of 0.05% type II collagenase (Worthington) in Dulbecco's modified Eagle's medium (DMEM) heated to 37°C was injected at <2–3 mL/min and refluxed. The liver was removed from the body. Then, the gallbladder, bile ducts, and hepatic veins were removed before the liver was minced. The minced livers were digested with 0.2% type II collagenase (Worthington) supplemented with 2.4 U/mL Dispase in DMEM for 60 min at 37°C. The digested livers were passed through 18‐G and 21‐G needles five times each.

#### 4.5.5 Lungs

The main bronchial tube was removed, and all lung lobes were minced. Minced lungs were digested with 0.2% type II collagenase (Worthington) for 60 min at 37°C. The digested lungs were passed through 18‐G and 21‐G needles five times each.

#### 4.5.6 Intestinal muscle layer

The mucosal layer was removed to mince the muscle layer. Minced muscle layers were digested with 2 mg/mL type II collagenase (Worthington) supplemented with 2 mg/mL BSA, 2 mg/mL trypsin inhibitor, and 0.3 mg/mL ATP in Ca‐free Hanks for 30 min at 37°C. Digested muscle layers were passed through an 18‐G needle five times and further digested with 0.1 mg DNaseI and 1 U/mL dispase for 10 min at 37°C. The digested slurries were passed through a 21‐G needle five times.

#### 4.5.7 Intestinal mucosal layer

The muscle layer was removed and the mucosal layer was cut into 5‐mm pieces. The mucosal layers were incubated in RPMI supplemented with 20 mM HEPES, 10 mM EDTA, and 5% fetal bovine serum (FBS) adjusted to pH 7.4 for 30 min at 37°C. The mucosal layers were washed three times with Ca‐free Hanks' solution and minced. Minced mucosal layers were digested with 2 mg/mL type II collagenase (Worthington) in Ca‐free Hanks' solution for 30 min at 37°C. Digested mucosal layers were passed through an 18‐G needle five times and digested with 0.1 mg DNaseI and 1 U/mL dispase for 10 min at 37°C. The digested slurries were passed through a 21‐G needle five times.

Each tissue slurry prepared was diluted with PBS, filtered through a 100 μm cell strainer, and then through a 40 μm cell strainer (BD Biosciences, Franklin Lakes, NJ, USA). The slurries were centrifuged × 1000*g* for 5 min at 25°C and the supernatants discarded before the cells were resuspended in washing buffer containing 2.5% FBS in PBS and stained for cell sorting. The reagents used for cell surface staining are listed in Table S8. Cell sorting was performed using a FACSAria II instrument (BD Biosciences). The gating strategy is presented in Figure S1.

### 4.6 Cell culture and differentiation assay

MSCs were isolated via flow cytometry and seeded onto Matrigel‐coated (CORNING, Corning, NY, USA) 8‐well chambers (Lab‐Tek Chamber slide, Thermo Fisher Scientific) or a μ‐Slide 8‐well plate (Ibidi, Gräfelfing, Germany). MSCs were cultured to 90% confluency in growth medium (DMEM supplemented with 20% FBS, 1% penicillin–streptomycin, and 2.5 ng/mL basic fibroblast growth factor (bFGF) (Hygieia Bioscience, Osaka, Japan)) at 37°C in 5% CO_2_ and 3% O_2_.

MSCs were differentiated into fibroblasts or osteoblasts for 4 days using differentiation medium (DMEM supplemented with 5% horse serum and 1% penicillin–streptomycin) containing 5 ng/mL rhTGF‐β1 (#7754‐BH, R&D Systems, Minneapolis, MN, USA) or 200 ng/mL rhBMP‐4 (#314‐BP, R&D Systems), respectively to assess their differentiation potential. For adipogenesis, cells were replaced with induction medium (DMEM supplemented with 10 μg/mL insulin, 0.25 μM dexamethasone, 0.5 mM IBMX, and 10% FBS) and cultured for 3 days. The culture medium was then carefully replaced with maintenance medium (DMEM supplemented with 10 μg/mL insulin and 10% FBS) and incubated for 4 days until fat droplets increased. Cells were fixed with 4% PFA for 5 min and washed with PBS. Then, αSMA staining was performed on cells that differentiated into fibroblasts. Cells were permeabilized with 0.1% Triton X‐100 in PBS, blocked, and incubated with monoclonal anti‐α‐smooth muscle actin Cy3‐conjugated antibody. They were subsequently washed with PBS and incubated with DAPI (DOJINDO) before observation. Cells that differentiated into osteoblasts underwent alkaline phosphatase (ALP) staining using the Blue‐Color TM AP Staining Kit (System Biosciences). They were incubated with an ALP substrate solution for 15 min under light‐shielded conditions and washed three times with PBS to stop the substrate reaction before microscopic observation. Lipid droplets were stained using an Adipocyte Fluorescent Staining kit for cells that differentiated into adipocytes (Cosmo Bio, Tokyo, Japan). The cells were incubated at 20°C for 30 min with an adipocyte staining solution (BODIPY®) pre‐warmed to 37°C. Next, nuclear stain (Hoechst 33258) was added before the cells were incubated at 20°C for 30 min and washed twice with PBS. ALP staining images were captured using a microscope (Optiphot‐2; Nikon) equipped with a CCD camera (DXM1200C; Nikon). Fluorescent images were captured using a DM6000FS fluorescence microscope (Leica Microsystems). Finally, αSMA‐, BODIPY‐, and ALP‐positive areas were quantified using the WinROOF2015 software (Mitani Corporation, Tokyo, Japan).

### 4.7 SHG imaging and 2D fourier transform (2D‐FT) analysis

Keratocan belongs to SLRPs that contribute to collagen fibrillogenesis (Kalamajski & Oldberg, 2010). Therefore, we examined the collagen structure of the EDL tendons of *Kera* KO mice using an SHG microscope capable of analyzing the distribution and orientation of collagen fibers. Here, muscles were fixed in 4% PFA for 30 min and stored in PBS at 4°C. SHG images were captured at an excitation wavelength of 870 nm. The polarization state of the laser light was converted to a circular polarization plate (WPQ10M‐850; Thorlabs Inc., NJ, USA) to cancel the polarization dependence of the SHG light. A depth of 35 μm from the surface of all tendon samples was captured. The FWHM of the angular spectrum on the acquired SHG images was processed as previously described (Hase et al., 2021) to quantify the distribution of the fiber orientation angle.

### 4.8 In vitro contractile force measurement

As *Kera* is specifically expressed at sites important for force transmission, and its deficiency alters collagen fiber orientation, we investigated the contractile properties of skeletal muscles in *Kera* KO mice. Measurements were performed as previously described (Minato et al., 2021). The EDL muscles were used for this experiment as it is necessary to attach the tendons at both ends of the muscle to the device to measure ex vivo muscle tension. The isolated EDL muscle was secured using a 5–0 silk suture at the distal and proximal tendons in an experimental chamber filled with mammalian Ringer solution containing (mM) NaCl (137), NaHCO_3_ (24), glucose (11), KCl (5), CaCl_2_ (2), MgSO_4_ (1), NaH_2_PO_4_ (1), and tubocurarine chloride (0.025) adjusted to pH 7.4. The MyoDynamics Muscle Strip System (model 840MD; DMT, Hinnerup, Denmark), square pulse electrical stimulator (model CS200; DMT), and PowerLab 4/26 data acquisition system (ADInstruments, Dunedin, New Zealand) were used to measure the contractile force. Specific tetanic force was obtained by dividing the absolute maximum tetanic force by the physiological cross‐sectional area.

### 4.9 Bulk RNA‐seq and differential gene expression analysis

Total RNA was extracted from sorted cells using the RNeasy Micro kit (Qiagen). RNA‐seq libraries were constructed using the Smart‐seq v4 Ultra Low Input RNA Kit for Sequencing (Cat# 634888; TaKaRa) and the Nextera TX DNA Library Prep Kit for Illumina (Cat# FC‐131–1024; Illumina). Libraries were sequenced using Illumina NovaSeq 6000 (Illumina), according to the instructions. Paired‐end sequencing reads were aligned against the mouse reference genome (GRCm38) using DRAGEN Bio‐IT Platform (version 3.6.3, Illumina). The quantified read counts from each sample were combined into a count matrix, with each row representing a unique gene ID and each column representing the raw counts for each sample. Data analysis was performed using R (version 4.1.0, R Foundation for Statistical Computing, Vienna, Austria). The R “DESeq” package (version 1.32.0) (Love et al., 2014) was used to normalize the raw counts and calculate the *p*‐values. The fold‐change was log2 scaled, and the Benjamini–Hochberg method was used to correct the *p*‐values for multiple testing. The results of the GO analysis of genes common to MSCs of seven tissues were illustrated using the “clusterProfiler” package (Yu et al., 2012). Differentially expressed genes (DEGs) between MSCs of young and aged skeletal muscles were identified using transcripts with log2 Fold‐change >2.5 and a 5% false discovery rate cutoff for the thresholds. PCA was performed using iDEP (version 0.96, http://bioinformatics.sdstate.edu/idep). Heat maps were created using Heatmapper (Babicki et al., 2016).

### 4.10 Statistical analyses

All statistical analyses were performed using Prism (version 9.5.1; GraphPad Software, San Diego, CA, USA). The equality of variance between the groups was analyzed using the F‐test or Bartlett's test. Differences between the two groups were analyzed using a two‐sided unpaired Student's *t* test, and Welch's correction was applied when the variances between the groups were not equal. Differences between more than two groups were analyzed using one‐way analysis of variance (ANOVA), or Brown‐Forsythe and Welch ANOVA tests when variances between the groups were not equal. Differences were considered statistically significant when the *p* < 0.05.

## AUTHOR CONTRIBUTIONS

T. K. and A. U. designed the study and interpreted the data. T.K., M. I.‐U., Y.Y., K.M., N.K., T.C., and E.H. performed experiments and analyses. T.M., T.Y., K.H., S.I., and M.H. analyzed and interpreted the data. T.K. and A.U. prepared the manuscript. All the authors have read and approved the final version of the manuscript.

## CONFLICT OF INTEREST STATEMENT

The authors declare no competing financial interests.

## Supporting information


**Data S1:** Supporting Information.

Table S1:

Table S2:

Table S3:

Table S4:

## Data Availability

The RNA‐seq data that support the findings of this study are openly available in the database DDBJ at (https://www.ddbj.nig.ac.jp), reference number [DRR518256‐DRR518309]. All the data presented here are available from the corresponding authors upon reasonable request.
