# Supplementary material for: Tissue‐specific functions of MSCs are linked to homeostatic muscle maintenance and alter with aging
Source: Aging Cell. 2024 Sep 25;23(11):e14299. doi: 10.1111/acel.14299 (PMC11561651; doi:10.1111/acel.14299)
Supplement: Supplementary file 1 — Data S1: Supporting Information. [file ACEL-23-e14299-s001.pdf]

Supplemental Information

Tamaki Kurosawa et al.

Tissue-specific functions of MSCs are linked to homeostatic muscle maintenance and alter with aging

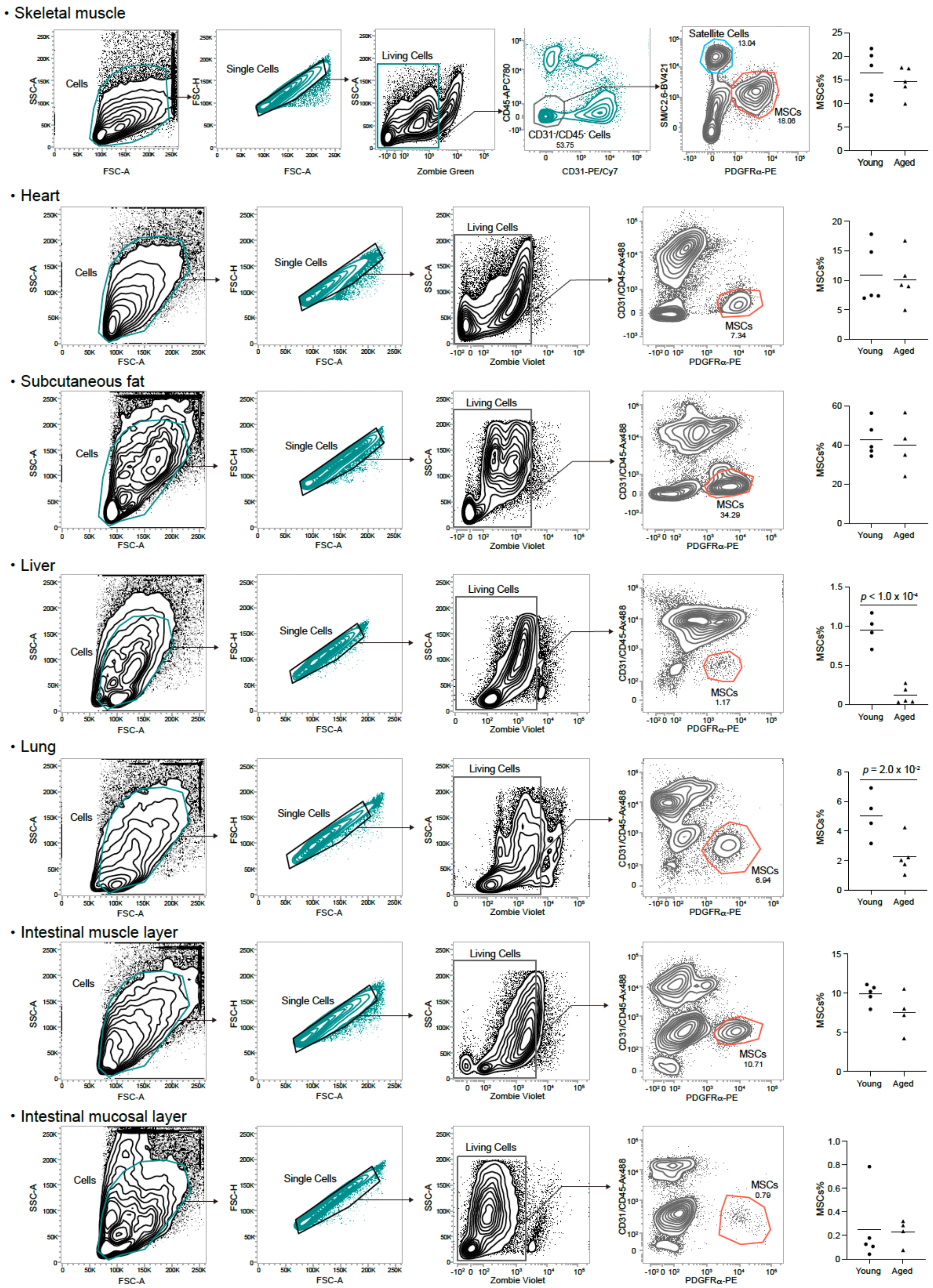

Supplemental Figure 1: Gating strategy for cell sorting and the percentages of MSCs in seven tissues.

For all seven tissues, orange frames refer to the gating of CD31$^-$/CD45$^-$/PDGFRα$^+$ MSCs. For skeletal muscle, a light blue frame refers to the gating of satellite cells. The numbers in the plots indicate the percentage of each cell in the live cells. The rightmost column shows the percentage of MSCs in the living cells in each tissue of young and aged mice.

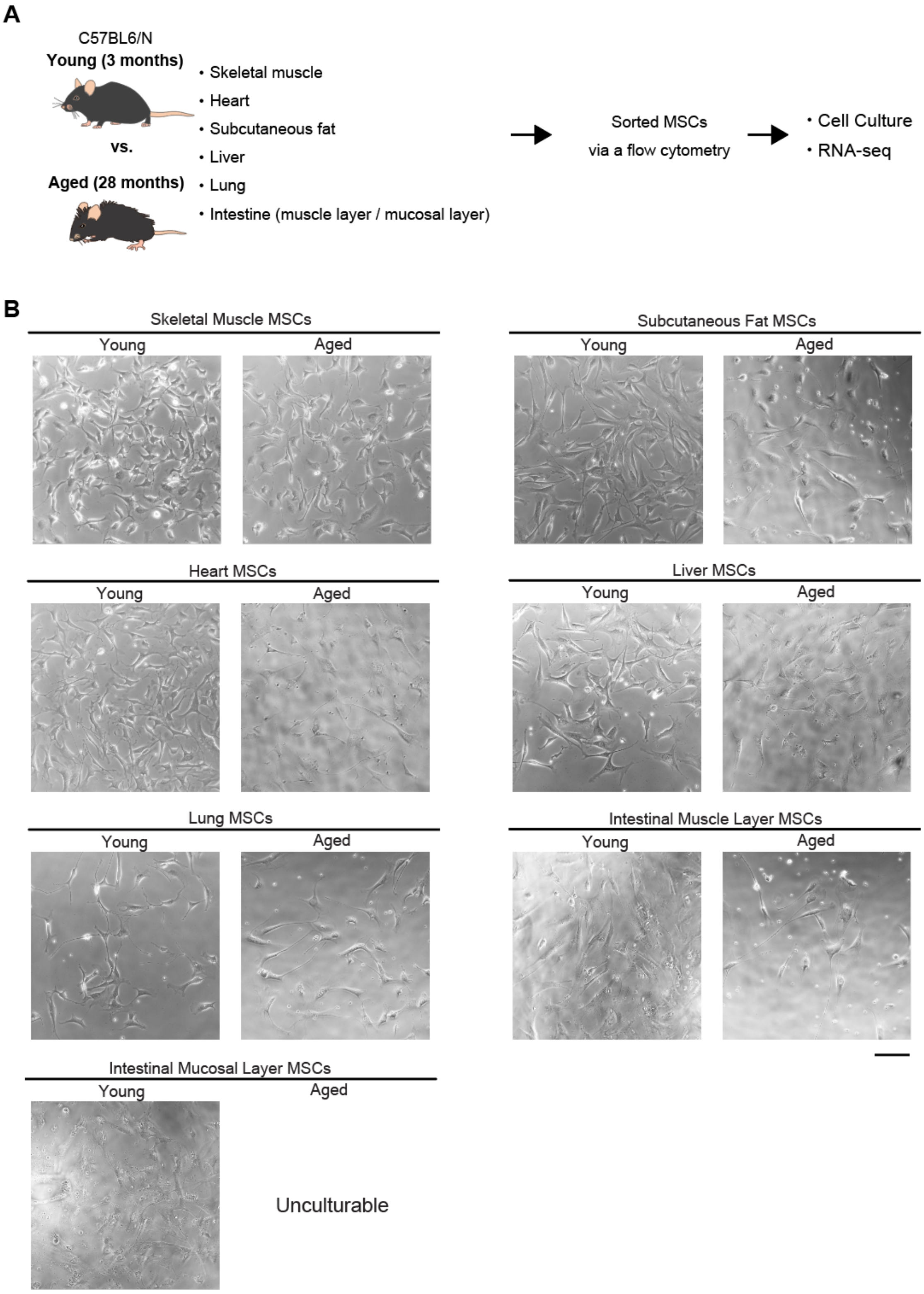

Supplemental Figure 2: The experimental scheme showing MSC isolation and morphology *in vitro*

A. The experimental scheme for RNA sequencing (RNA-seq) of MSCs from seven tissues belonging to six organs. **B**. Images of the cultured MSCs that were isolated from seven tissues of young and aged mice using flow cytometry. Scale bar = 100 μm.

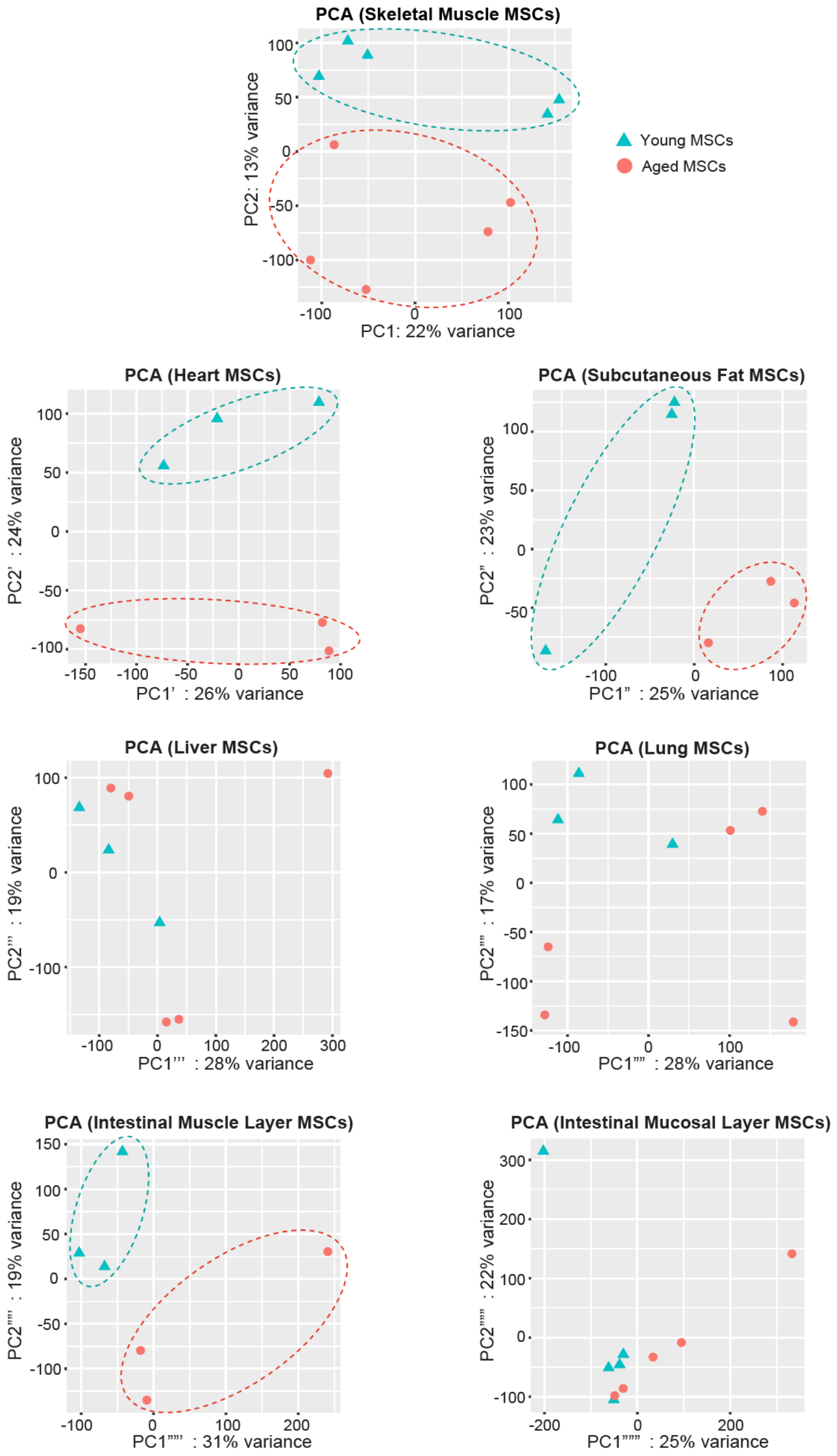

Supplemental Figure 3: PCA plots of MSCs in each tissue.

PCA analysis for RNA-seq of MSCs from seven tissues of young and aged mice. This results from a re-analysis of the data presented in Fig. 4A, separated by each tissue. The dashed circles represent clusters for young (light blue) and aged (pink) samples.

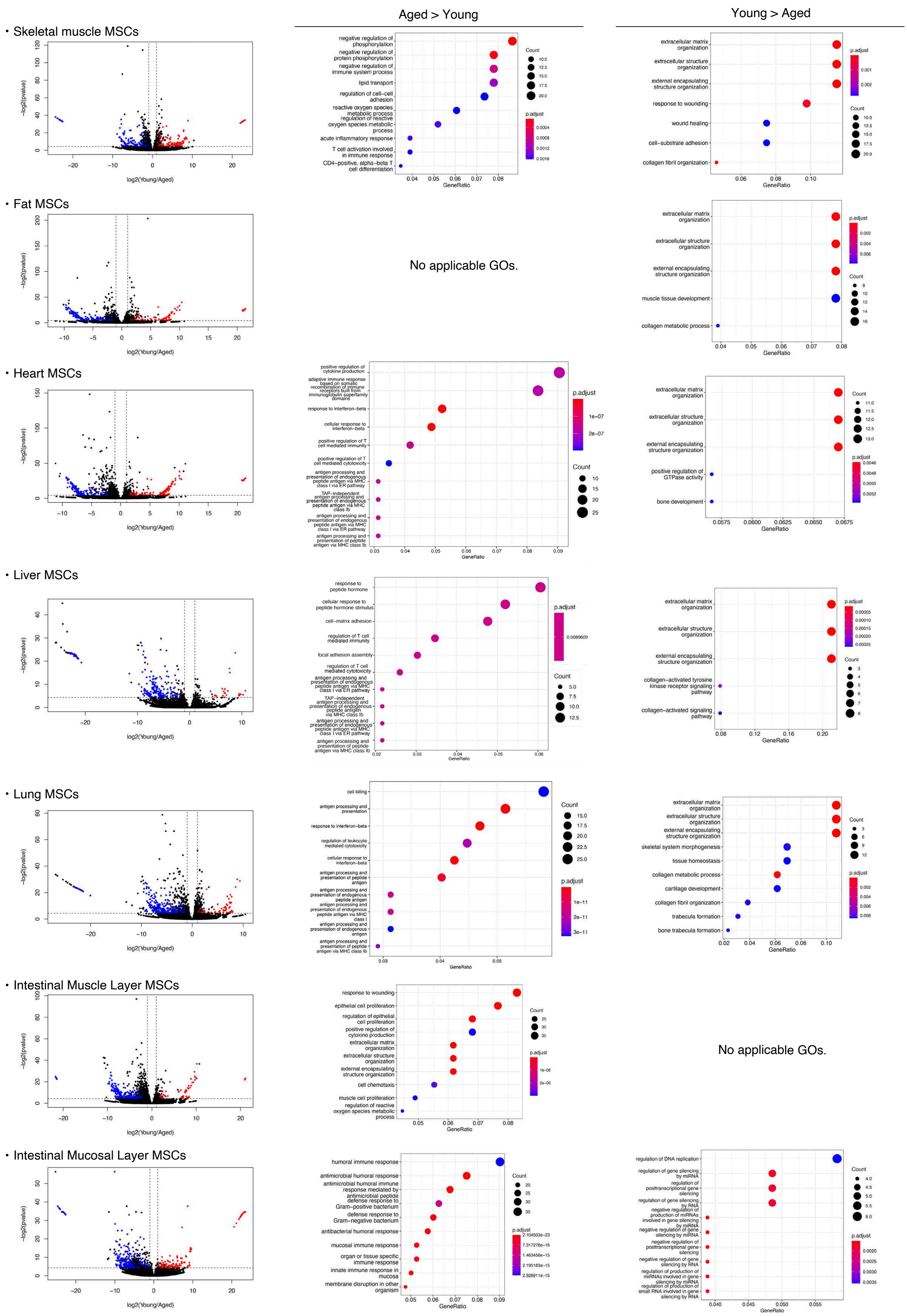

Aged > Young
Young > Aged
• Skeletal muscle MSCs
−log2(pvalue)
log2(Young/Aged)
negative regulation of phosphorylation
negative regulation of protein phosphorylation
negative regulation of immune system process
lipid transport
regulation of cell−cell adhesion
reactive oxygen species metabolic process
regulation of reactive oxygen species metabolic process
acute inflammatory response
T cell activation involved in immune response
CD4−positive, alpha−beta T cell differentiation
GeneRatio
Count
p.adjust
extracellular matrix organization
extracellular structure organization
external encapsulating structure organization
response to wounding
wound healing
cell−substrate adhesion
collagen fibril organization
• Fat MSCs
No applicable GOs.
extracellular matrix organization
extracellular structure organization
external encapsulating structure organization
muscle tissue development
collagen metabolic process
• Heart MSCs
positive regulation of cytokine production
adaptive immune response based on somatic recombination of immune receptors built from immunoglobulin superfamily domains
response to interferon−beta
cellular response to interferon−beta
positive regulation of T cell mediated immunity
positive regulation of T cell mediated cytotoxicity
antigen processing and presentation of endogenous peptide antigen via MHC class I via ER pathway
TAP−independent antigen processing and presentation of endogenous peptide antigen via MHC class Ib
antigen processing and presentation of endogenous peptide antigen via MHC class I via ER pathway
antigen processing and presentation of peptide antigen via MHC class Ib
extracellular matrix organization
extracellular structure organization
external encapsulating structure organization
positive regulation of GTPase activity
bone development
• Liver MSCs
response to peptide hormone
cellular response to peptide hormone stimulus
cell−matrix adhesion
regulation of T cell mediated immunity
focal adhesion assembly
regulation of T cell mediated cytotoxicity
antigen processing and presentation of endogenous peptide antigen via MHC class I via ER pathway
TAP−independent antigen processing and presentation of endogenous peptide antigen via MHC class Ib
antigen processing and presentation of endogenous peptide antigen via MHC class I via ER pathway
antigen processing and presentation of peptide antigen via MHC class Ib
extracellular matrix organization
extracellular structure organization
external encapsulating structure organization
collagen−activated tyrosine kinase receptor signaling pathway
collagen−activated signaling pathway
• Lung MSCs
cell killing
antigen processing and presentation
response to interferon−beta
regulation of leukocyte mediated cytotoxicity
cellular response to interferon−beta
antigen processing and presentation of peptide antigen
antigen processing and presentation of endogenous peptide antigen
antigen processing and presentation of peptide antigen via MHC class I
antigen processing and presentation of endogenous antigen
antigen processing and presentation of peptide antigen via MHC class Ib
extracellular matrix organization
extracellular structure organization
external encapsulating structure organization
skeletal system morphogenesis
tissue homeostasis
collagen metabolic process
cartilage development
collagen fibril organization
trabecula formation
bone trabecula formation
• Intestinal Muscle Layer MSCs
response to wounding
epithelial cell proliferation
regulation of epithelial cell proliferation
positive regulation of cytokine production
extracellular matrix organization
extracellular structure organization
external encapsulating structure organization
cell chemotaxis
muscle cell proliferation
regulation of reactive oxygen species metabolic process
No applicable GOs.
• Intestinal Mucosal Layer MSCs
humoral immune response
antimicrobial humoral response
antimicrobial humoral immune response mediated by antimicrobial peptide
defense response to Gram−positive bacterium
defense response to Gram−negative bacterium
antibacterial humoral response
mucosal immune response
organ or tissue specific immune response
innate immune response in mucosa
membrane disruption in other organism
regulation of DNA replication
regulation of gene silencing by miRNA
regulation of posttranscriptional gene silencing
regulation of gene silencing by RNA
negative regulation of production of miRNAs involved in gene silencing by miRNA
negative regulation of gene silencing by miRNA
negative regulation of posttranscriptional gene silencing
negative regulation of gene silencing by RNA
regulation of production of miRNAs involved in gene silencing by miRNA
regulation of production of small RNA involved in gene silencing by RNA

Supplemental Figure 4: Age-related changes in the MSCs of seven tissues.

Volcano plots were used to visualize the DEGs of young and aged MSCs in seven tissues. The red and blue dots represent genes whose expression is significantly downregulated and upregulated with aging, respectively. Genes with BaseMean >100, log2FC >1, and adj. *p*-value <0.05 were extracted as DEGs. The representative GO terms enriched in each MSC DEG of the seven tissues are shown.

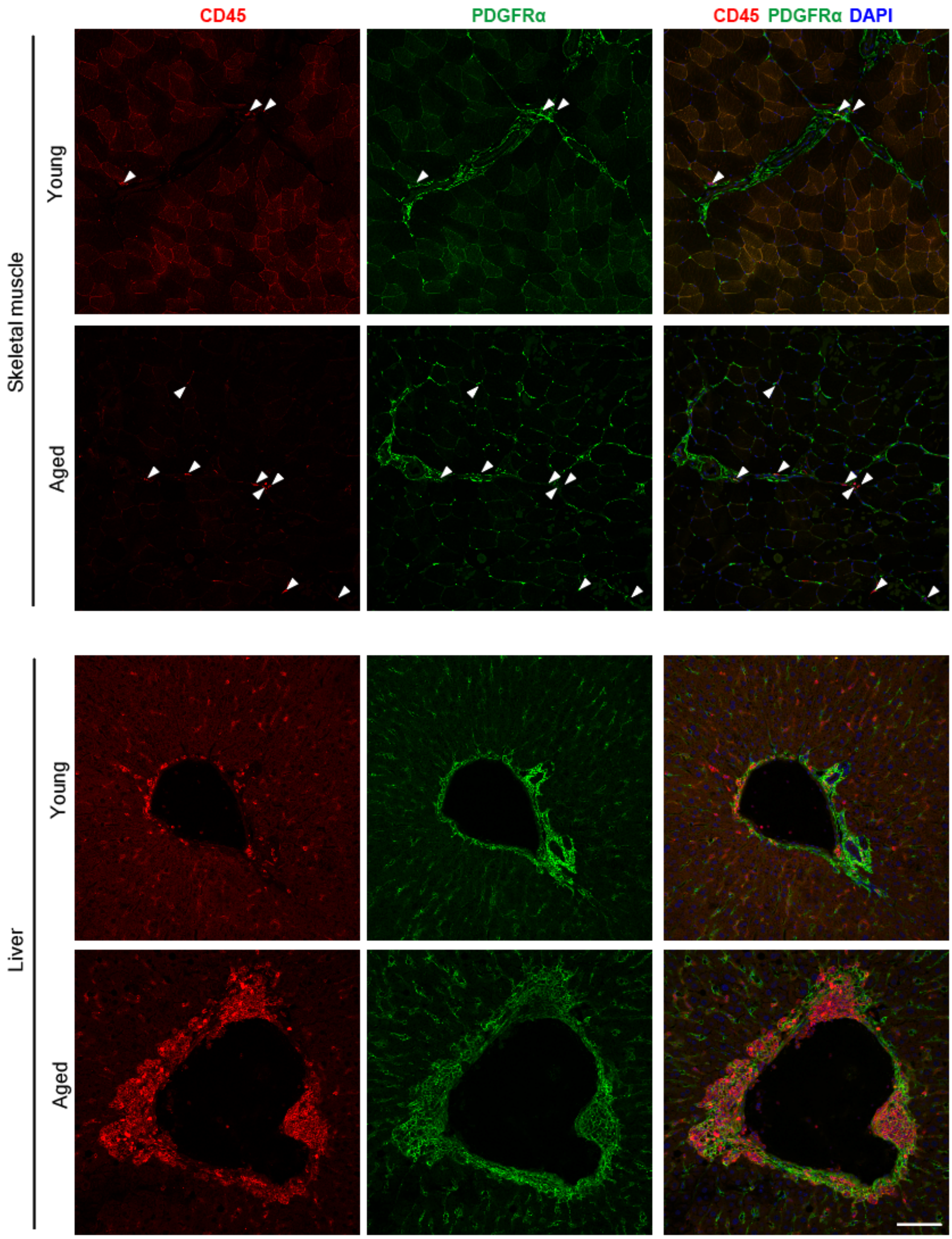

Supplemental Figure 5: CD45-positive immune cells exist abundantly and close to MSCs in aged tissues. Fluorescence immunostaining images of skeletal muscle and the liver for CD45 (red) and PDGFRα (green) visualization. Samples were counterstained with DAPI (blue). Arrows indicate $CD45^{+}$ cells in skeletal muscle images. Scale bar = 100 μm.

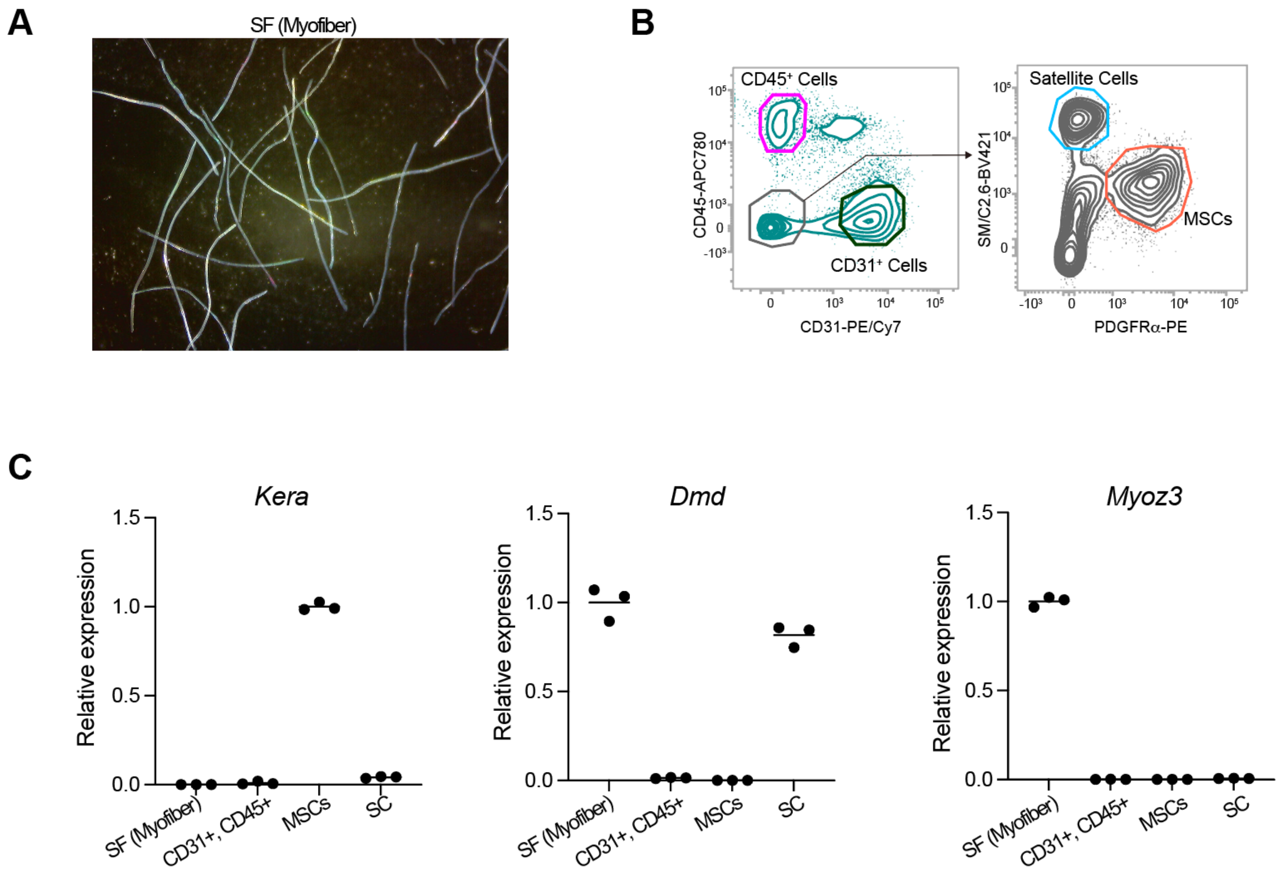

Supplemental Figure 6: *Kera* expression was highly specific to MSCs and undetectable in other cell types within the muscle tissue.

A. Images of the collected single fibers that ware analyzed as myofiber. **B.** FACS plot showing the cell fractions collected as CD31+, CD45+, MSCs, or Satellite cells. **C.** The quantified expression levels of *Kera, Dmd,* and *Myoz3*.SF: Single fiber, $CD31^{+}$, $CD45^{+}$: CD31 or CD45-positive cells, MSCs: PDGFRα-positive MSCs, SC: satellite cells. *Dmd* was used as a marker gene for SF and SC. *Myoz3* was used as a marker gene for SF.

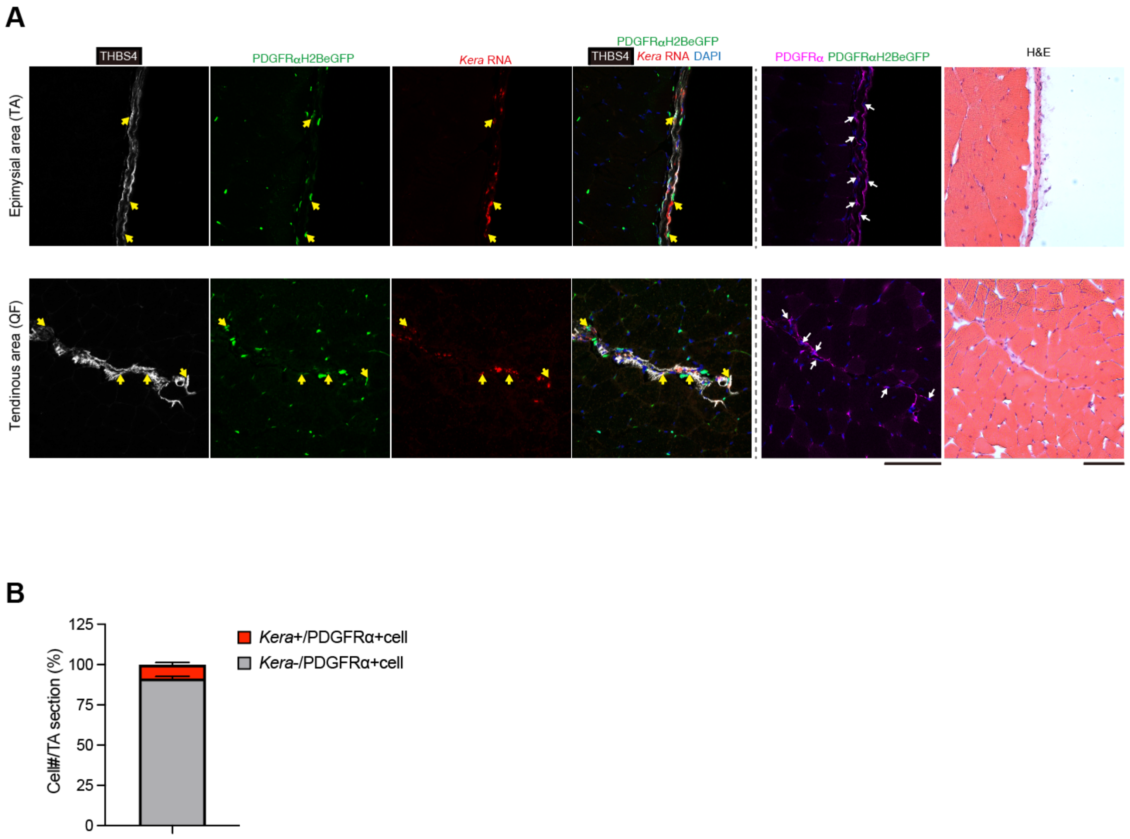

Supplemental Figure 7: *Kera* is expressed in MSCs in specific regions of skeletal muscle.

A. *In situ* hybridization on muscle sections from young PDGFRαH2BeGFP mice with *Kera* (red) probe followed with immunostaining to THBS4 (white) and GFP (green) and counterstained with DAPI (blue). Images of serial sections stained with PDGFRα antibody (magenta) followed with H&E staining are shown in the same row to the right side of the dashed lines. Yellow arrows indicate *Kera*+/PDGFRαH2BeGFP+ nuclei. White arrows indicate PDGFRα+ cells. TA: tibialis anterior, QF: quadriceps femoris. Scale bar = 100 µm. **B.** Percentages of *Kera*+/PDGFRαH2BeGFP+ nuclei in total PDGFRαH2BeGFP+ nuclei per TA section.

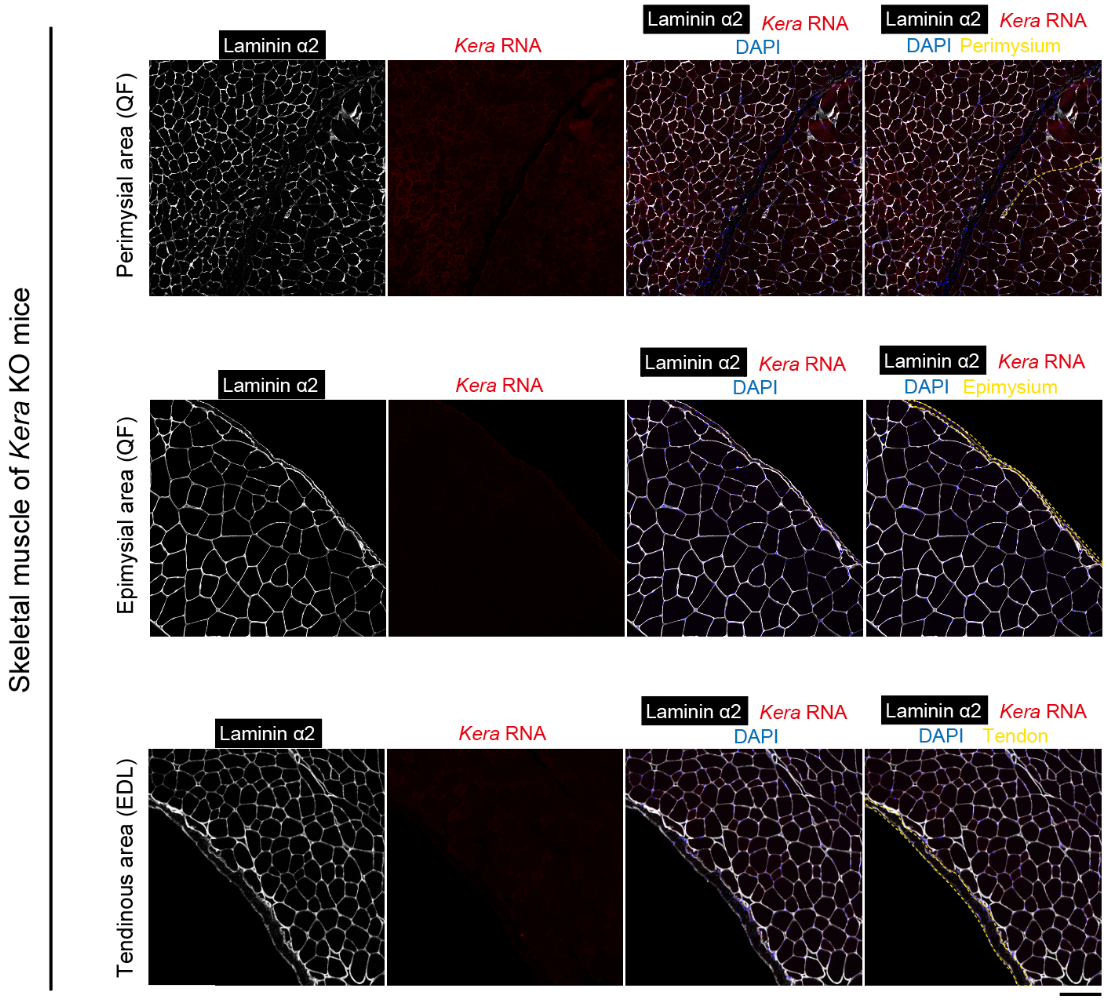

Supplemental Figure 8: *Kera* is not expressed in the muscle interstitium of *Kera* KO mice.

In situ hybridization of skeletal muscle tissue sections obtained from *Kera* KO mice using a *Kera* (red) probe. Yellow dashed lines indicate perimysial, epimysial, or tendinous areas. The sections were counterstained with laminin α2 (white) and DAPI (blue). QF: quadriceps femoris, EDL: Extensor digitorum longus. Scale bar = 100 µm.

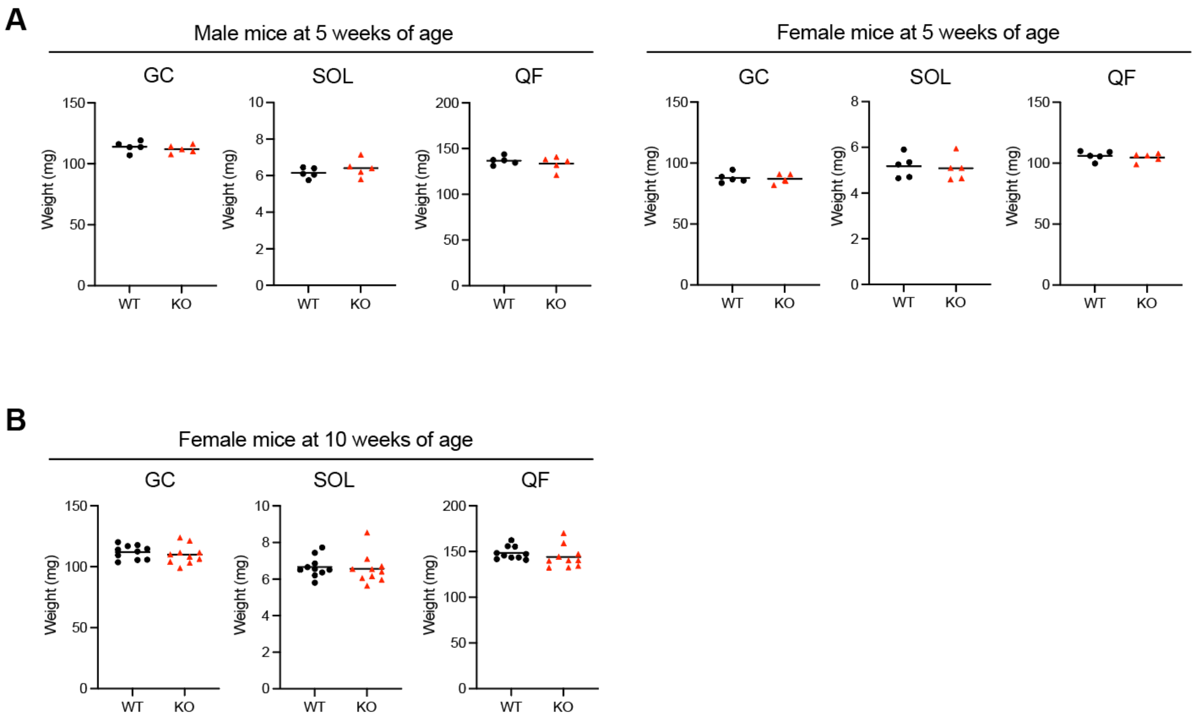

Supplemental Figure 9: Muscle weight measurements of *Kera* KO mice of different week-olds and sexes.
A. The muscle weight of the *Kera* KO or wildtype (WT) littermates was measured at 5 weeks of age. n = 5 (*Kera* KO) and n = 5 (WT). **B.** The muscle weight of the female *Kera* KO or wildtype (WT) littermates was measured at 10 weeks of age. n = 10 (*Kera* KO) and n = 10 (WT). GC: gastrocnemius, SOL: soleus, QF: quadriceps femoris.

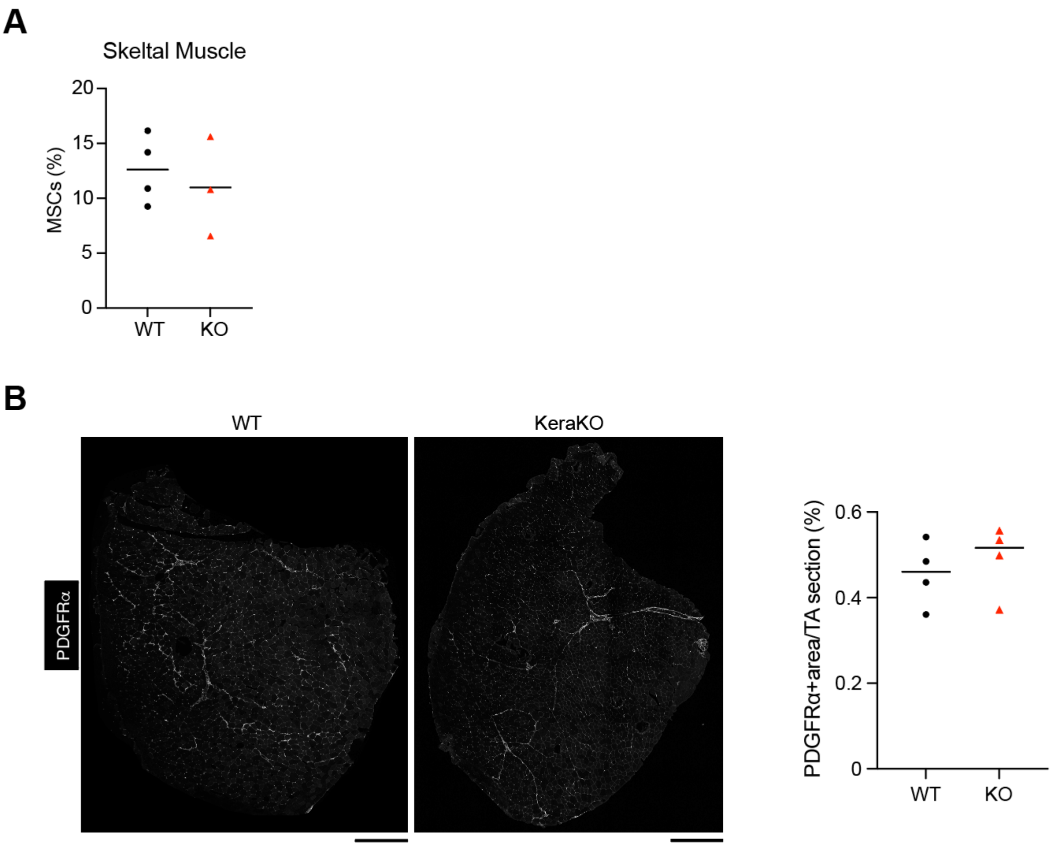

Supplemental Figure 10: There is no difference in the number of PDGFRα+ cells in skeletal muscle tissue between WT and *Kera* KO mice.

A. The percentage of MSCs in the living cells in hind limb muscle tissue of WT and *Kera* KO mice analyzed by FACS. **B.** Fluorescence immunostaining images of TA sections of WT and *Kera* KO mice for PDGFRα (white) visualization. The percentages of PDGFRα+ area per TA section are shown in the graph. Scale bar = 500 μm.

Supplemental Table 1: The list of genes to visualize the tissue specificity of MSCs

This is a list of TPM values for the data shown as a heat map in Figure 4B. Genes with TPM values >150 and expression ratios >1 in log2FC to those in MSCs from other tissues' MSCs were selected as genes that refract MSC tissue specificity. This data is provided separately as a CSV file.

Supplemental Table 2: The list of extracted genes whose expression in skeletal muscle MSCs was downregulated with aging.

This is a list of data shown as a volcano plot in Figure 5A. Genes with a variation of BaseMean >100, log2FC >1, and adj. *p*-value <0.05 were extracted as DEGs. The samples shown in Fig. 4 were used for the analysis. This data is provided separately as a CSV file.

Supplemental Table 3: The list of extracted genes whose expression in skeletal muscle MSCs was upregulated with aging.

This is a list of data shown as a volcano plot in Figure 5A. Genes with a variation of BaseMean >100, log2FC >1, and adj. *p*-value <0.05 were extracted as DEGs. The samples shown in Fig. 4 were used for the analysis. This data is provided separately as a CSV file.

Supplemental Table 4: The list of TPM value of ten genes with the highest expression levels in young muscle MSCs.

This is a list of TPM values for the data shown as a heat map in Figure 5B. The genes with variations of log2FC >2.5 and adj. *p*-value <0.05 were selected as DEGs. The samples shown in Fig. 4 were used for the analysis. This data is provided separately as a CSV file.

Supplemental Table 5: The list of sexes of samples used in the analysis.

Figure#	Sample name	Sex
**Fig.1**		
	Skeletal Muscle	male
	Heart	male
	Subcutaneous Fat	male
	Lung	male
	Liver	male
	Intestine	male
**Fig.2**		
	Skeletal Muscle	male
	Heart	male
	Subcutaneous Fat	male
	Lung	male
	Liver	male
	Intestine	male
**Fig.3**		
A-C	Skeletal Muscle MSCs	female
A-C	Heart MSCs	male
A-C	Subcutaneous Fat MSCs	male
A-C	Lung MSCs	female
A-C	Liver MSCs	female
A-C	Intestine MSCs	female
**Fig.4**		
A-C	Skeletal Muscle MSCs Young1	female
A-C	Skeletal Muscle MSCs Young2	female
A-C	Skeletal Muscle MSCs Young3	missing
A-C	Skeletal Muscle MSCs Young4	female
A-C	Skeletal Muscle MSCs Young5	female
A-C	Liver MSCs Young1	female
A-C	Liver MSCs Young2	missing
A-C	Liver MSCs Young3	female
A-C	Lung MSCs Young1	female
A-C	Lung MSCs Young2	missing
A-C	Lung MSCs Young3	female
A-C	Heart MSCs Young1	male
A-C	Heart MSCs Young2	male
A-C	Heart MSCs Young3	male
A-C	Fat MSCs Young1	male
A-C	Fat MSCs Young2	male
A-C	Fat MSCs Young3	male
A-C	Int Muscle MSCs Young1	female
A-C	Int Muscle MSCs Young2	female
A-C	Int Muscle MSCs Young3	female
A-C	Int Mucosal MSCs Young1	female
A-C	Int Mucosal MSCs Young2	female
A-C	Int Mucosal MSCs Young3	female
A-C	Int Mucosal MSCs Young4	female
A-C	Int Mucosal MSCs Young5	missing
A-C	Skeletal Muscle MSCs Aged1	female
A-C	Skeletal Muscle MSCs Aged2	female
A-C	Skeletal Muscle MSCs Aged3	male
A-C	Skeletal Muscle MSCs Aged4	missing
A-C	Skeletal Muscle MSCs Aged5	missing
A-C	Liver MSCs Aged1	female
A-C	Liver MSCs Aged2	female
A-C	Liver MSCs Aged3	female
A-C	Liver MSCs Aged4	missing
A-C	Liver MSCs Aged5	missing
A-C	Lung MSCs Aged1	female
A-C	Lung MSCs Aged2	female
A-C	Lung MSCs Aged3	female
A-C	Lung MSCs Aged4	missing
A-C	Lung MSCs Aged5	missing
A-C	Heart MSCs Aged1	male
A-C	Heart MSCs Aged2	male
A-C	Heart MSCs Aged3	male
A-C	Fat MSCs Aged1	male
A-C	Fat MSCs Aged2	male
A-C	Fat MSCs Aged3	male
A-C	Int Muscle MSCs Aged1	missing
A-C	Int Muscle MSCs Aged2	female
A-C	Int Muscle MSCs Aged3	missing
A-C	Int Mucosal MSCs Aged1	missing
A-C	Int Mucosal MSCs Aged2	female
A-C	Int Mucosal MSCs Aged3	female
A-C	Int Mucosal MSCs Aged4	female
A-C	Int Mucosal MSCs Aged5	missing

Figure#	Sample name	Sex
**Fig.5**		
A, B	Same samples as Fig.4	
C	Skeletal Muscle	female
D	Skeletal Muscle	female
E	Skeletal Muscle	male
**Fig.6**		
A-H	Skeletal Muscle	male

**Suppl.Fig.1**		
	Representative data of Fig.3 and 4	
**Suppl.Fig.2**		
B	Representative data of Fig.3	
**Suppl.Fig.3**		
	Same samples as Fig.4	
**Suppl.Fig.4**		
	Same samples as Fig.4	
**Suppl.Fig.5**		
	Skeletal Muscle	male
	Liver	male
**Suppl.Fig.6**		
A-C	Skeletal Muscle	female
**Suppl.Fig.7**		
A, B	Skeletal Muscle	male
**Suppl.Fig.8**		
	Skeletal Muscle	male
**Suppl.Fig.9**		
A	Skeletal Muscle	male, female
B	Skeletal Muscle	female
**Suppl.Fig.10**		
A, B	Skeletal Muscle	male

Supplemental Table 6: Antibodies used for IHC

Primary Antibody	Dilution	Supplier
Rat anti mouse CD31 (Clone: MEC13.3)	1:300	Biosciences,<br>Cat.# 550274
Goat polyclonal anti mouse PDGFRα	1:400	R&D<br>Cat.# AF1062
Griffonia simplicifolia IB_4 Isolectin conjugated with Alexa Fluor^{TM} 647	1:200	Invitrogen ^{TM}<br>REF: 132450
Rabbit anti mouse PGP9.5	1:500	UltraClone Limited
Rat anti mouse c-kit (Clone: ACK4)	1:500	Invitrogen ^{TM}
Mouse anti a-smooth muscle actin Cy3®-conjuagted (Clone: 1A4)	1:400	SIGMA<br>Cat. # C6198
Rat anti-laminin α2 antibody	1:400	Santa Cruz Biotechnology<br>Cat. # sc-59854
Rat monoclonal anti mouse Thrombospondin-4 (Clone: 893655)	1:200	R&D<br>Cat. # MAB7860
Chicken anti GFP	1:500	Abcam<br>Cat. # ab13970

Secondary Antibody	Dilution	Supplier
Cy3®-conjugated anti goat IgG	1:1000	Jackson laboratory
Alexa Fluor® 647-conjugated anti goat IgG	1:1000	Jackson laboratory
Alexa Fluor® 647-conjugated anti rat IgG	1:1000	Jackson laboratory
Alexa Fluor® 488-conjugated anti rabbit IgG	1:1000	Jackson laboratory
Cy3®-conjugated anti rat IgG	1:1000	Jackson laboratory
Alexa Fluor® 647-conjugated donkey anti rat IgG	1:1000	Jackson laboratory
Goat Anti-Chicken IgY H&L Alexa Fluor® 488-conjugated	1:1000	Invitrogen ^{TM}<br>REF: A-11039
DAPI	1:2000	DOJINDO
BODIPY®	This product is included in the kit.	Cosmo Bio Co., Ltd. Adipocyte Fluorescent Staining kit, Code No. AK19F
Lipidye II	1μM	Funakoshi Co., Ltd.<br>REF: FDV-0027

Supplemental Table 7: Primers used for PCR

**Gene**	**Direction**	**Sequence**
Hsbp1	Forward	5’-CAAGACCATGCAGGACATCAC-3’
	Reverse	5’-AGGTCAGCGATATTCTTCTCCA-3’
Cmas	Forward	5'-CAAAGGCATCCCACTGAAGA-3'
	Reverse	5'-CCCACACACTCTGGAAGACC-3'
Kera	Forward	5’-TCAGTTGTGGTCCGTGAATG-3’
	Reverse	5’-CCCGAATCAATGCTAACCTG-3’
Dmd	Forward	5'-GGAAGAAGTAGAGGACTGTTATG-3'
	Reverse	5'-AGGTCTAGGAGGCGTTTTCC-3'
Myoz3	Forward	5'-TGGCAGCAGAAGTCACACTC-3'
	Reverse	5'-AGTTCCAAGCCACTGAAGGAC-3'

Hsbp1 (in Fig. 5C) and *Cmas* (in Fig. 5D and Suppl. Fig. 6C) were used as endogenous control genes.

Supplemental Table 8: Antibodies used for cell isolation

For Skeletal muscle	**Dilution**	**Supplier**
Rat anti mouse CD31- PE/Cyanine 7 conjugated (Clone: 390)	1:300	BioLegend,<br>Cat. # 102418
Rat anti mouse CD45- APC-eFluor 780 conjugated (Clone: 30-F11)	1:300	eBioscience,<br>Cat. # 47-0451-82
Goat polyclonal anti mouse PDGFRα PE-conjugated	10 mL/sample	R&D,<br>Cat. # FAB1062P
Biotinylated rat anti mouse satellite cells (Clone: SM/C-2.6)	1:300	No commercialization
Brilliant Violet 421™ streptavidin	1:300	BD Horizon™,<br>Cat. # 563259
Zombie Green™ Dye	1:500	BioLegend,<br>Cat. # 423111

For Heart, Subcutaneous fat, Liver, Lung, and Intestine	**Dilution**	**Supplier**
Rat anti mouse CD31- Alexa Fluor® 488 conjugated (Clone: 390)	1:300	BioLegend,<br>Cat. # 102414
Rat anti mouse CD45- Alexa Fluor® 488 conjugated (Clone: 30-F11)	1:300	BioLegend,<br>Cat. # 103122
Goat polyclonal anti mouse PDGFRα PE-conjugated	10 mL/sample	R&D,<br>Cat. # FAB1062P
Zombie Violet™ Dye	1:500	BioLegend,<br>Cat. # 423113
